# Overcoming the effects of false positives and threshold bias in graph theoretical analyses of neuroimaging data

**DOI:** 10.1016/j.neuroimage.2015.05.011

**Published:** 2015-09

**Authors:** M. Drakesmith, K. Caeyenberghs, A. Dutt, G. Lewis, A.S. David, D.K. Jones

**Affiliations:** a Cardiff University Brain Research Imaging Centre (CUBRIC), School of Psychology, Cardiff University, Park Place, Cardiff CF10 3AT, UK; b Neuroscience and Mental Health Research Institute (NMHRI), School of Medicine, Cardiff University, Maindy Road, Cardiff CF24 4HQ, UK; c School of Psychology, Faculty of Health Sciences, Australian Catholic University, 115 Victoria Parade, Melbourne, VIC 3065, Australia; d Institute of Psychiatry, King's College London, 16 De Crespigny Park, London SE5 8AF, UK; e Division of Psychiatry, Faculty of Brain Sciences, University College London, Charles Bell House, 67-73 Riding House Street, London W1W 7EJ, UK

## Abstract

Graph theory (GT) is a powerful framework for quantifying topological features of neuroimaging-derived functional and structural networks. However, false positive (FP) connections arise frequently and influence the inferred topology of networks. Thresholding is often used to overcome this problem, but an appropriate threshold often relies on *a priori* assumptions, which will alter inferred network topologies.

Four common network metrics (global efficiency, mean clustering coefficient, mean betweenness and smallworldness) were tested using a model tractography dataset. It was found that all four network metrics were significantly affected even by just one FP. Results also show that thresholding effectively dampens the impact of FPs, but at the expense of adding significant bias to network metrics.

In a larger number (*n* = 248) of tractography datasets, statistics were computed across random group permutations for a range of thresholds, revealing that statistics for network metrics varied significantly more than for non-network metrics (i.e., number of streamlines and number of edges). Varying degrees of network atrophy were introduced artificially to half the datasets, to test sensitivity to genuine group differences. For some network metrics, this atrophy was detected as significant (*p* < 0.05, determined using permutation testing) only across a limited range of thresholds.

We propose a multi-threshold permutation correction (MTPC) method, based on the cluster-enhanced permutation correction approach, to identify sustained significant effects across clusters of thresholds. This approach minimises requirements to determine a single threshold *a priori*. We demonstrate improved sensitivity of MTPC-corrected metrics to genuine group effects compared to an existing approach and demonstrate the use of MTPC on a previously published network analysis of tractography data derived from a clinical population.

In conclusion, we show that there are large biases and instability induced by thresholding, making statistical comparisons of network metrics difficult. However, by testing for effects across multiple thresholds using MTPC, true group differences can be robustly identified.

## Highlights

• False positives (FPs) have profound effects on graph theoretical (GT) analysis.
• Thresholding can reduce the effects of FPs, but introduces its own bias.
• Statistics on GT metrics are highly variable across thresholds.
• Group effects can be robustly detected using a new permutation method (MTPC).
• MTPC is more sensitive and robust than the existing area-under-curve (AUC) method.

## Introduction

In the past decade, interest in brain connectivity among neuroimaging researchers has grown substantially. There is now a wide array of techniques for inferring brain connectivity from in vivo brain imaging. Structural connectivity derived from diffusion-weighted imaging (DWI) is well established and several approaches for deriving axonal pathways have been developed (Basser et al., 1994; Behrens et al., 2007; Conturo et al., 1999; Dell'acqua et al., 2010; Descoteaux et al., 2009; Jansons and Alexander, 2003; Jeurissen et al., 2011; Jones, 2008; Jones et al., 1999; Parker and Alexander, 2005; Tournier et al., 2008; Tuch et al., 2004; Wedeen et al., 2005). Functional connectivity, which is defined as statistical relationships between neural signals (Sporns, 2007), can be derived from electroencephalography (EEG) and magnetoencephalography (MEG) (Baccalá and Sameshima, 2001; Granger, 1969; Kamiński et al., 2001; Lachaux et al., 1999; Nolte et al., 2004; Nunez et al., 1997; Schreiber, 2000; Stam et al., 2007; Walter, 1968; Wibral et al., 2009) and functional magnetic resonance imaging (fMRI) (Baumgartner et al., 2000; Biswal et al., 1995; Chuang et al., 1999; Ngan and Hu, 1999; Worsley et al., 2005).

The increasing interest in brain connectivity has led to new questions being asked about how to characterise properties of networks, beyond examining piecemeal components. Graph theory (GT) is a powerful mathematical framework for quantifying topological properties of networks. A graph is comprised of nodes (or vertices) and edges. Nodes are defined using brain regions and edges defined by anatomical or functional connections derived using the methods described above. In recent years, the approach has emerged as a useful tool for characterising functional and structural brain networks (Bullmore and Sporns, 2009; Filippi et al., 2013; Guye et al., 2010; Rubinov and Sporns, 2010; Sporns and Tononi, 2007; Stam and Reijneveld, 2007). This type of analysis (referred to as graph theoretical analysis or network analysis) moves away from the traditional neuroimaging approach of examining individual components of the brain, such as cortical regions or white-matter tracts, and moves towards characterising regional or global structure of networks. This can be particularly valuable in the study of clinical disorders of non-focal degeneration (e.g., Alzheimer's disease) or systemic dysfunction (e.g., schizophrenia) and for understanding information processing differences in healthy and diseased brains. Network analysis has been utilised to examine network changes in several clinical disorders (see Griffa et al., 2013; Petrella, 2011; Xia and He, 2011 for reviews) in resting-state networks (Achard and Bullmore, 2007; Salvador et al., 2005; van den Heuvel et al., 2008; Wang et al., 2010). Some recent studies have also used network analysis to investigate event-related or task-related changes in the topology of functional networks (Kida and Kakigi, 2013; Markett et al., 2014; J.X. Wang et al., 2013).

Additionally, the edges can be weighted by the original connectivity indices, or by other quantitative anatomical or physiological measurements. This is common for structural connectivity metrics; where edges can initially be defined from tractography analysis, and then weighted by a microstructural measurement, such as fractional anisotropy or myelination (e.g., van den Heuvel et al., 2010).

### Sources of error in inferred brain connectivity

While network analysis is a powerful tool for understanding the topology of brain networks, there is a critical issue in that these measures are sensitive to the errors in the inferred edges, both in the presence of edges and in their derived weights. False positives (FPs) and false negatives (FNs) can arise when inferring both functional and structural networks and, depending on the processing parameters employed, there is always a trade-off between the two. In the context of tractography, an FP refers to finding a streamline between two regions, which, either connect regions when there is no true connection, or between two regions where there is a true connection, but takes a pathway which is not true to the underlying anatomy. An FN is the failure to generate streamlines between regions for which there is a true connection.

In tractography-derived assessments of structural connectivity, there are several sources of error: The well-established approach of diffusion tensor imaging (DTI) is unable to resolve fibre orientation distributions (FODs) adequately in regions of crossing fibres (Alexander, 2006; von dem Hagen and Henkelman, 2002). Streamlines derived with DTI tractography are likely to terminate or choose only one dominant direction in such regions. DTI tractography is therefore prone to a large number of FNs. In contrast, using other FOD reconstruction methods, such as constrained spherical deconvolution (CSD) (Tournier et al., 2008) can lead to spurious peaks in the FOD due to low angular resolution, low SNR or poorly characterised fibre response functions (Parker et al., 2013a,b). This will result in more erroneous streamline trajectories and more FPs.

Another source of error is the difficulty in determining the true trajectory of streamlines emerging from regions of merging or kissing fibres (e.g., Fillard et al., 2011). In such cases, streamlines may be ‘diverted’ to a path that is not consistent with the underlying anatomy. Another source of error is an inherent bias due to distance, curvature and branching. In longer, curving or branching pathways, the accumulated error in tract propagation means that the termination criteria is more likely to be reached than in short, straight fibres (Jones, 2010). This bias will alter the topology of the inferred network considerably. Furthermore, more general confounds in network analysis can arise irrespective of the connectivity measure employed (see Fornito et al., 2013; Papo et al., 2014 for reviews). There are multiple strategies for deriving the nodes of the graph, which can significantly alter the topology of the inferred networks (see Stanley et al., 2013 for a review). Also the way edge weights are derived will lead to biases in the network topology particular to the measurement employed.

Test–retest reliability of network metrics has been examined in a number of recent studies (see Telesford et al., 2013, for a review). For example, Vaessen et al. (2010) found that many network metrics from tractography data were relatively stable across acquisitions with different gradient amplitudes and number of diffusion encoding directions, although clustering coefficient was slightly more variable.

Subsequent studies have also suggested the network metrics from tractography are highly reproducible across acquisitions (Bassett et al., 2011; Dennis et al., 2012; Owen et al., 2013). However, there are studies that indicate otherwise. Bastiani et al. (2012) showed that graph metrics are biased by the chose of tractography algorithm and parameters, while Buchanan et al. (2014) found that the reliability of graph metrics was dependent on the positioning of the seed points and the FOD model used.

More generally, the approach of test–retest reliability assumes a random variation in the FP/FN rates in the inferred networks. Some of the sources of FPs described above give rise to systematic biases in FP/FN rates that do not necessarily change across acquisitions, and therefore will not affect test–retest reliability. For example, a subject with particularly high curvature of the uncinate fasciculus will be less easily reconstructed than for other subjects (Jones, 2010), but the test–retest reliability of the inferred topology from this subject will not be affected. In such cases there is an interaction between subject-specific variability (e.g., anatomy) and the stochastic processes that contribute to FPs. This type of interaction cannot be ruled out by only examining test–retest reliability.

### Thresholding

One approach is to threshold the network prior to network analysis. The motivation for this is that small edge weights are assumed to arise from FPs rather than genuine anatomical connections. However, there are a number of issues with this approach: (1): The threshold chosen is often arbitrary. The appropriate threshold should be able to suppress FPs adequately. However, the FP rate is typically unknown and is specific to the unique methodology used to infer the network; and (2) thresholding increases the chance of FNs in the network. This can have equally detrimental effects on network metrics because of the potential removal of small but functionally relevant connections. There is also the issue that increasing threshold will reduce the networks to the core network components, which, when comparing between groups, may remove distinguishing network features, resulting in non-significant group differences.

For this reason, many researchers opt not to apply thresholds to graphs and to compute GT metrics on unthresholded weighted graphs (e.g., van den Heuvel et al., 2010). This approach relies on the assumption that, provided the edges are weighted, small (and presumably false) edge weights will have inconsequential effects on the computed metrics. However this assumption is contentious in the literature and recently shown to be incorrect (Ginestet et al., 2011, 2014).

Some approaches have been proposed to reduce the arbitrariness of threshold selection. Van Wijk et al. (2010) reviewed a number of criteria used for the determination of the appropriate threshold. Many studies that used statistical indices of connectivity (e.g., correlation coefficients) simply choose a threshold that is deemed statistically significant (Chen et al., 2008; Ferrarini et al., 2009). Other studies have used a minimal level of network density or degree (the number of edges connecting each node) as the criterion for thresholding (Meunier et al., 2009; van den Heuvel et al., 2009). Bassett et al. (2006) used a number of criteria to determine an appropriate threshold. One is the false discovery rate (FDR) correction method (Benjamini and Hochberg, 1995) to estimate the FP rate and keep this to below 5%. Bassett et al. (2006) also constrained the threshold using the average node degree, which must be maintained at a minimum number of connected nodes. Some other studies chose a threshold where a minimum mean degree has to be maintained across all groups (Bartolomei et al., 2006; Bernhardt et al., 2011; de Haan et al., 2009; Dimitriadis et al., 2009; Ferri et al., 2008). This however assumes that node degree is the same between groups. This will be particularly problematic in ‘disconnection syndromes’ or cases of neurodegeneration, where this assumption is likely to be invalid. In such cases, fixing node degree will bias the network structure and hence any comparisons of network metrics. Choosing different criteria for different groups also causes problems, as other network metrics will be dependent on this property. This will increase the risk of erroneous inference of significant group differences.

In all these cases, *a priori* assumptions of core network properties need to be made to judge the criteria for thresholding correctly. To overcome these issues, a number of studies have opted to compute network metrics across a range of thresholds in a more agnostic fashion (Hosseini et al., 2012; van den Heuvel et al., 2009). Although this provides a more complete picture of network differences, it makes the results more ambiguous and difficult to interpret. Another approach is to summarise the network metrics across a range of thresholds, typically computing the area under the curve (AUC) of the resultant profile of network metrics across thresholds (Cao et al., 2013; Hosseini et al., 2012; Rubinov et al., 2009; Zhang et al., 2011). This approach is attractive as it is easy to compute and considers network topology across many thresholds with reduced reliance on *a priori* assumptions. However, the range of thresholds to sum across should be chosen with care. Inclusion of low thresholds is likely to include effects from FPs while inclusion of very high thresholds will include measurements of highly disconnected networks that are not reflective of the true connectome. Also there can be a failure to detect true group effects if the effect only manifests in a limited range of thresholds.

Langer et al. (2013) explored the effects of thresholding on statistical inference in network metrics derived from EEG functional connectivity. They showed that thresholding affected the ability to detect differences between groups. Summarising network metrics, such as computing AUCs, prior to testing for group differences, can therefore be problematic as group effects can become diluted, particularly if the effect is visible in only a narrow range of thresholds (Ginestet et al., 2011). Langer et al. (2013) proposed applying multiple thresholds, computing network metrics for each sample and performing inferential statistics across samples for each group. The authors point out that this approach is problematic due to the non-independence between networks at different thresholds. They also investigated an alternative approach computing group-permuted metrics, where connectivity metrics were permuted between groups and a null distribution of networks was computed. A number of recent papers have promoted the use of permutation testing to perform statistical tests on network metrics given the highly complex and, often, non-linear operations involved in their computation (Hosseini et al., 2012; Langer et al., 2013; Simpson et al., 2013; Zalesky et al., 2010). Langer et al. (2013) further proposed the use of permutation testing across thresholds for more reliable testing on network metrics. However, to our knowledge, this has yet to be implemented.

### Aims

The main aim of the present study was to determine, systematically, the extent to which false positive streamline reconstructions impact on the network metrics of structural networks derived using tractography, and to examine the effects of thresholding to overcome this issue. Our aims were threefold: (1) to study the effects of FPs on a simplified model dataset and to examine the effects of thresholds on the network metrics themselves; (2) to examine the effects of thresholding on inferential statistics of network metrics on a large dataset; and (3) to propose and demonstrate a multi-threshold permutation correction (MTPC) approach for improved sensitivity to genuine group effects with minimal *a priori* assumptions. The MTPC method is then applied to a previously published graph theoretical analysis of structural networks in a clinical population.

## General method

### MRI acquisition

MRI scans from 248 healthy participants (mean age = 19.6 y, 86 male, 162 female) obtained from the Avon Longitudinal Study of Children and Parents (ALSPAC) birth cohort (Golding et al., 2001) were used. Informed consent was obtained prior to scanning and the study was performed with ethical approval from the local ethics review boards. MRI data were acquired on a 3 T General Electric HDx MRI system (GE Medical Systems, Milwaukee, WI) using an eight-channel receive-only head RF coil. A cardiac-gated HARDI EPI protocol was used with 60 gradient orientations (Jones et al., 1999) and 6 unweighted (b = 0 s/mm^2^) images. TE = 87 ms, b = 1200 s/mm^2^, 60 slices, slice thickness = 2.4 mm, FoV = 230 × 230 mm, acquisition matrix = 96 × 96, with a 2.4 mm^3^ isotropic resolution. Each slice was up-sampled within-plane to 1.8 mm^2^, giving a voxel-size = 1.8 × 1.8 × 2.4 mm.

### Tractography

HARDI data were analysed in ExploreDTI v4.8.2 (Leemans et al., 2009). Data were corrected for motion, eddy current and field inhomogeneity distortions prior to tractography (Leemans and Jones, 2009; Wu et al., 2008). Whole-brain tractography was performed using the damped Richardson–Lucy algorithm (Dell'acqua et al., 2010), This is a modified spherical harmonic method which is more robust to spurious peaks in the FOD than the standard CSD method (Tournier et al., 2007). Seed points were arranged in a 3 × 3 × 3 mm grid in white matter, step size = 1 mm, angle threshold = 45°, length threshold = 20–500 mm, FOD threshold = 0.05, *β* = 1.77, *λ* = 0.0019, *η* = 0.04, number of iterations = 200 (see Dell'acqua et al., 2010 for details of these parameters).

### Connectivity matrices

**Fig. 1:**
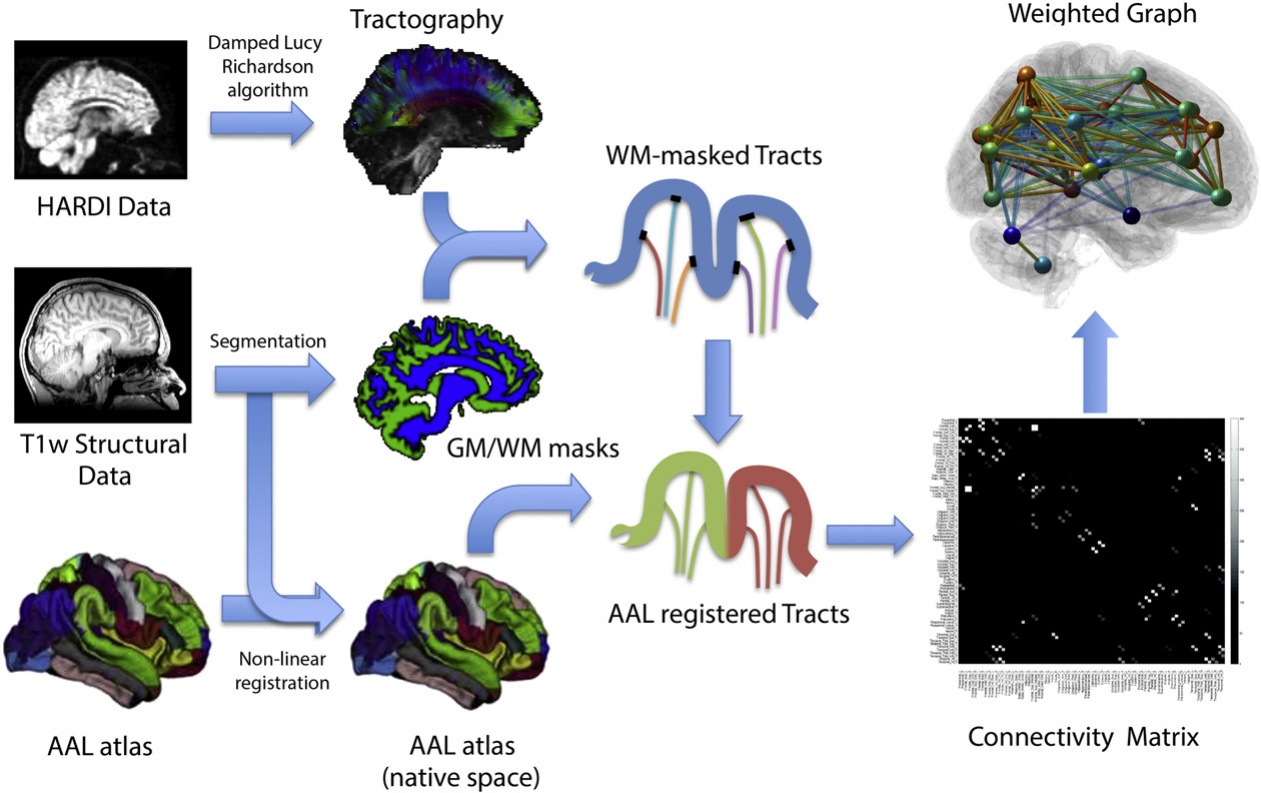
Flowchart of derivation of connectivity matrices from tractography data.

Fig. 1 shows a flowchart for the process of obtaining connectivity matrices. The Automated Anatomical Labelling (AAL) atlas (Tzourio-Mazoyer et al., 2002) was registered to the HARDI data using a non-linear transformation (Klein et al., 2010). The AAL atlas is commonly used to derive nodes in network analyses of neuroimaging data. The streamline termination points were co-registered to each AAL region. The numbers of streamlines connecting each pair of AAL regions were aggregated into a 116 × 116 connectivity matrix.

### Network metrics

Four commonly used network measures of global network topology were computed throughout this work: Global efficiency, mean clustering coefficient, mean betweenness and smallworldness. Comprehensive descriptions and equations for these metrics can be found in Rubinov and Sporns (2010). These metrics were chosen because: (1) they are all commonly used in network neuroimaging analyses; (2) they all measure distinct features of network topology; and (3) they are not necessarily inter-correlated with each other. In all cases, the network metrics were computed from weighted networks. The edges were weighted by the proportion of streamlines relative to the total number of streamlines. All network metrics were computed using the Brain Connectivity Toolbox (Rubinov and Sporns, 2010).

1. Global efficiency (Latora and Marchiori, 2001) is a measure of network integration. It is formally the inverse of the characteristic path length, which is the mean minimum (geodesic) distance required to travel from one node to another. It represents how efficiently information can be transmitted across the network.
2. Mean clustering coefficient (Onnela et al., 2005; Opsahl and Panzarasa, 2009; Watts and Strogatz, 1998) is a measure of functional segregation. More specifically, it is the tendency of the network to organise into functionally distinct clusters, with numerous and strong edges within clusters and few and weak edges between clusters. Formally, the clustering coefficient of a node is the ratio of the sum of the weights across all complete triangles around the node, to the number of edges connecting the node.
3. Mean betweenness centrality (Freeman, 1979) is a measure of centrality. Betweenness of a node is defined as the fraction of all shortest paths in the network that pass through a given node. This measures the importance of nodes to overall network integrity (Tijms et al., 2013; van den Heuvel et al., 2010). Formally, betweenness of a node is the sum of ratios of shortest paths traversing the node, versus the number of shortest paths in the whole network.
4. Smallworldness (Humphries and Gurney, 2008; Watts and Strogatz, 1998) is characterised by a high degree of clustering but each node can be reached easily by a low path length. It is typically used as a measure of ‘wiring-efficiency’ and is a structure found in many naturally occurring systems. Formally, smallworldness is the ratio of the normalized clustering coefficient vs. the normalized path length. The normalised versions of these two measurements are the original measure, normalised to the equivalent measure from a random network with the same density.

In addition to these metrics, some node-level metrics were also tested (see Supplementary material S1).

## Experiment 1a — effects of FPs in an model dataset

In this experiment, a ‘model’ dataset was created and regarded as a ‘ground truth’ network. FPs were added and the effects on network metrics quantified.

### Method

#### Creating the model dataset

**Fig. 2:**
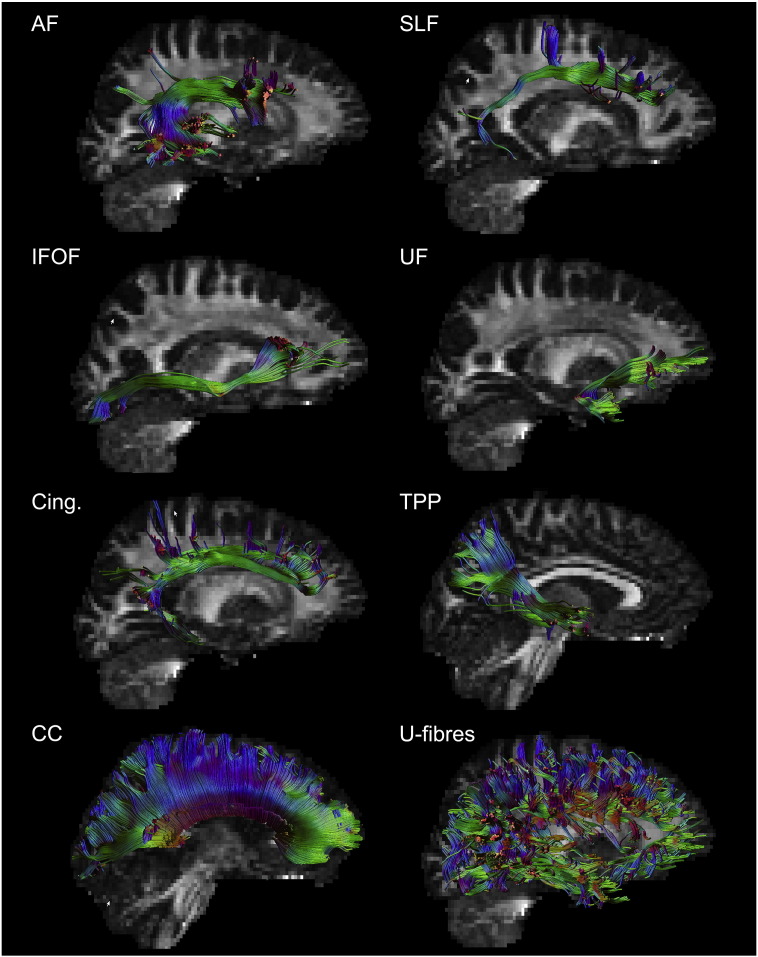
Selected bundles for construction of ‘ground truth’ model network. Arcuate fasiculus (AF) superior longitudinal fasiculus (SLF) inferior longitudinal fasciculus (ILF) uncinate fasciculus (UF) cingulum (Cing) temproparietal pathway (TPP) corpus calosum (CC) short-range U-fibres.

A model dataset was created from a single tractography dataset (male, 19 y) selected from the cohort described above. The streamlines were ‘pruned’ by removing any portions of the tracts that enter grey matter. Tracts traversing grey matter multiple times were split into separate tracts. This was done to eliminate FPs that can arise from erroneous trajectories taken when the streamline enters grey matter. Eight major fibre pathways between cortical regions were segmented manually within ExploreDTI (Fig. 2): Uncinate fasiculus (UF), superior longitudinal fasciculus (SLF), arcuate fasciculus (AF), inferior longitudinal fasciculus (ILF), inferior fronto-occipital fasciculus (IFOF), temporo-parietal pathway (TPP), cingulum (Cing) and corpus callosum (CC). Connecting AAL regions were identified by coregistering an inflated AAL atlas to the tract termination points. Cortical U-fibres were also included by identifying tracts connecting adjacent AAL regions. Anatomically inconsistent fibres, determined by visual inspection, were removed.

All intra-hemispheric fibres were restricted to the left hemisphere. The corpus callosum fibres were restricted to those connecting left and right homologues of each AAL region. Right hemisphere fibres were mirrored from the left hemisphere. The graph was based on an adjacency matrix mapping connectivity between all cortical AAL regions and initially weighted by the number of connecting streamlines associated with each pair of AAL regions.

#### Effects of false positives (FPs)

FPs were simulated by adding streamlines to edges of the network. Two different types of FPs were simulated to differentiate the effects of FPs creating new edges and FPs that overestimate existing edge weights. Both types of FPs can arise from streamlines taking anomalous trajectories through white matter. In addition to the main experiment, the effects of edge displacements were also investigated.

1. FPs creating new edges (FP-NEs): Streamlines were added to non-connecting pairs of nodes, creating new artefactual edges. Each FP that is added creates a unique edge, so the number of FPs is equal to the number of additional edges.
2. FPs added to existing edges (FP-EEs): Streamlines were added to existing edges only, so the number of edges in the network remains constant but the weighting of edges is overestimated.

**Fig. 3:**
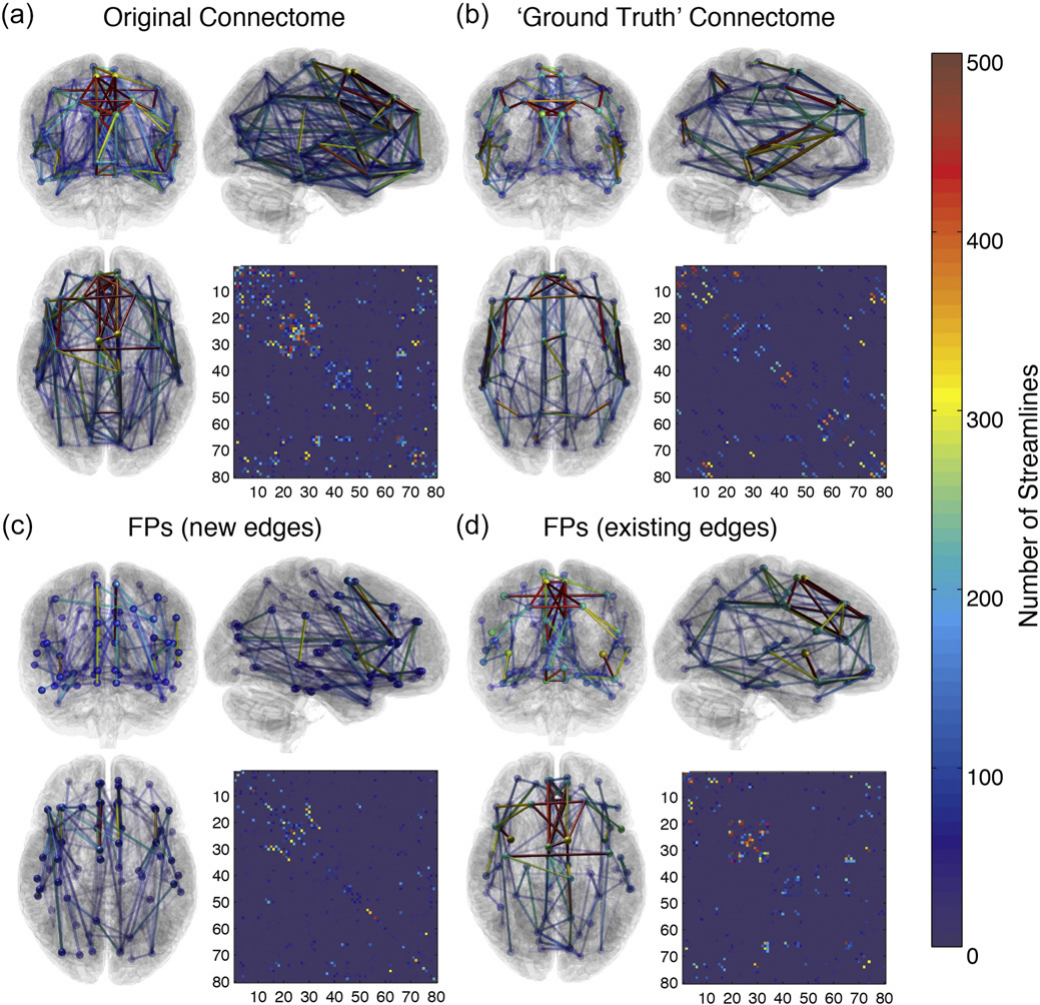
Visualisation of networks used in simulations. (a) is the original tractography dataset without any additional processing; (b) is the model network derived using manual segmentation of cortico-cortical pathways; (c) and (d) are the sets of FP-NEs and FP-EEs to be sampled, respectively. These were derived from the streamlines present in the original tractography dataset but not in the ground truth network.

FPs of each type were sampled from sets of FPs derived from the original ‘superset’ of streamlines, prior to manual segmentation and pruning, and excluding those streamlines included in the model dataset. The proportion of streamlines in each edge, relative to the total number in the set, is the probability of the streamline being sampled. This was done to create a distribution of FPs that reflects the FP rates encountered in a typical tractography analysis. This set was further divided into FP-NEs and FP-EEs in the model dataset, for the two experimental settings. These sets are visualised in Fig. 3.

In both cases, the number of FPs added to the network was varied between 0 and 20. The sampling of FPs was repeated across 100 randomisations. After adding FPs, the edge weights were normalised by the total number of streamlines to compensate for changes in network density. Network metrics were computed for each FP count and randomisation. The proportional change in each network metric relative to the ground truth network, and corresponding standard z-scores (i.e., the mean change normalised to its standard error) across randomisations was computed. Additional analysis of the effects of FPs on specific edges was also carried out (see Supplementary material S3).

### Results

**Fig. 4:**
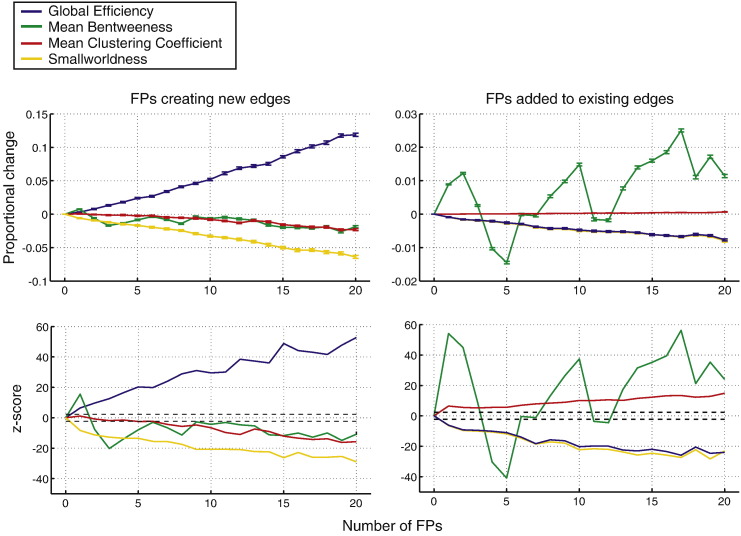
Impact of FP-NEs and FP-EEs on network metrics, both measured through proportional change and standard z-scores. Error bars on proportional error plots indicate standard error across iterations. Dotted lines on z-score plots indicate critical values of z for *p* < 0.01.

Results from our first experiment, examining the effects of FP-NEs and FP-EEs on network metrics are shown in Fig. 4. For FP-NEs, all metrics deviated significantly (at *p* < 0.01) from the model network with just one or more FPs, with the exception of clustering coefficient, which only deviated significantly with 6 or more FP-NEs. Global efficiency is systematically overestimated while other metrics are underestimated. Mean betweenness shows a non-monotonic change across thresholds at small FP-NE counts.

For FP-EEs, all metrics deviate from the model network with just one or more FPs. Clustering coefficient is overestimated while global efficiency and smallworldness are underestimated. Mean betweenness shows a very erratic non-monotonic profile of change across FP counts.

The range of proportional change at which significant differences occur are generally low (particularly for FP-EEs which are typically in the range of 0–0.03). However these are still in the range where significant topological network differences are identified in clinical populations (see Supplementary material S2).

## Experiment 1b — effects of edge displacement

In addition to the effects of FPs, we also tested the effects of displacement. This is where edges are moved from a correct pair of node to an incorrect pair. Although this is not a realistic scenario in the context of tractography analysis (except perhaps for very large errors in atlas registration), this type of error is interesting as there is no change in the number of edges (as in the FP-NE manipulation) or change in the edge weights (as in the FP-EE manipulation) and is influenced directly by false network topology.

### Method

Proportional change in the four GT metrics as a result of the varying number displacement was computed (0 to 20). Displacements were only made between adjacent streamline bundles, determined by a volumetric overlap of 30% or more in each pair of bundles.

### Results

**Fig. 5:**
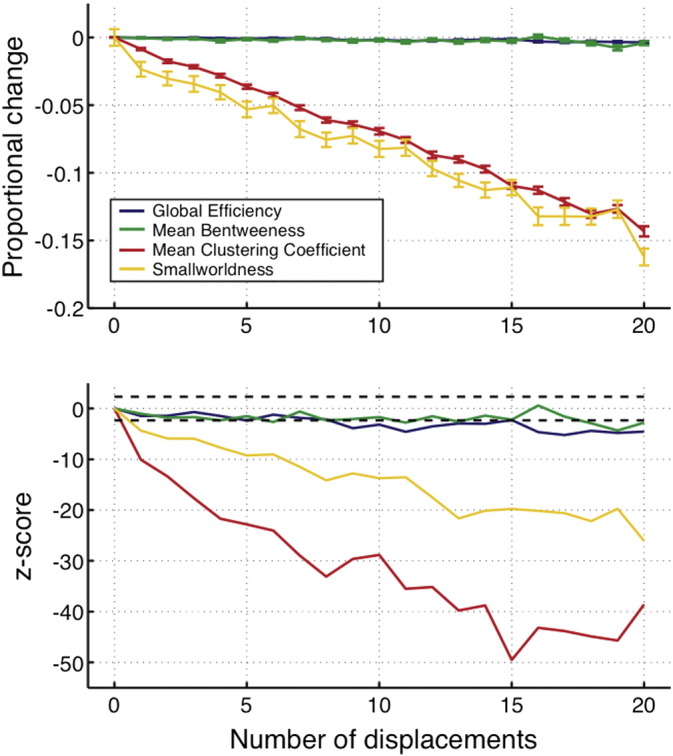
Impact of edge displacements on network metrics, both measured through proportional change and standard z-scores. Error bars on proportional error plots indicate standard error across iterations. Dotted lines on z-score plots indicate critical values of z for *p* < 0.01.

Results (Fig. 5) show that effects of global efficiency and mean betweenness are relatively unaffected, although close to the critical level of significance. Mean clustering coefficient and smallworldness however show strong negative effects. This shows that GT analysis is still vulnerable to topological errors, other than those involving changes in the number of edges or the edge weights.

## Experiment 1c — effects of thresholding in an model dataset

Using the original and altered networks from experiment 1a, the effects of thresholding on network metrics were quantified and their effectiveness at reducing the effects of FPs investigated.

### Method

To test the effects of thresholding on altered networks, network metrics were calculated across thresholds, *τ*, from 0 to 10 streamlines. Any edge with *τ* or less streamlines was removed. Thresholding was applied to all networks with all FP rates. Edges were weighted by the proportion of streamlines relative to the total number of streamlines. The overall *inter-threshold* effect was determined by computing the proportional change relative to the *unthresholded* ground truth network. To examine the *intra-threshold* effects on FPs (i.e., effect of thresholding on FPs, while normalising to the overall effects of thresholding on network metrics) the proportional change relative to the *thresholded* ground truth was computed. An additional analysis of the effects of thresholds on specific edges was also performed (see Supplementary material S3).

Note that for this experiment, only FP-EEs were investigated. As each FP-NE is unique to each edge, FP-NEs will be completely removed by thresholding. There will be no intra-threshold effects and inter-threshold effects would be the same as those shown by the ground-truth network.

### Results

**Fig. 6:**
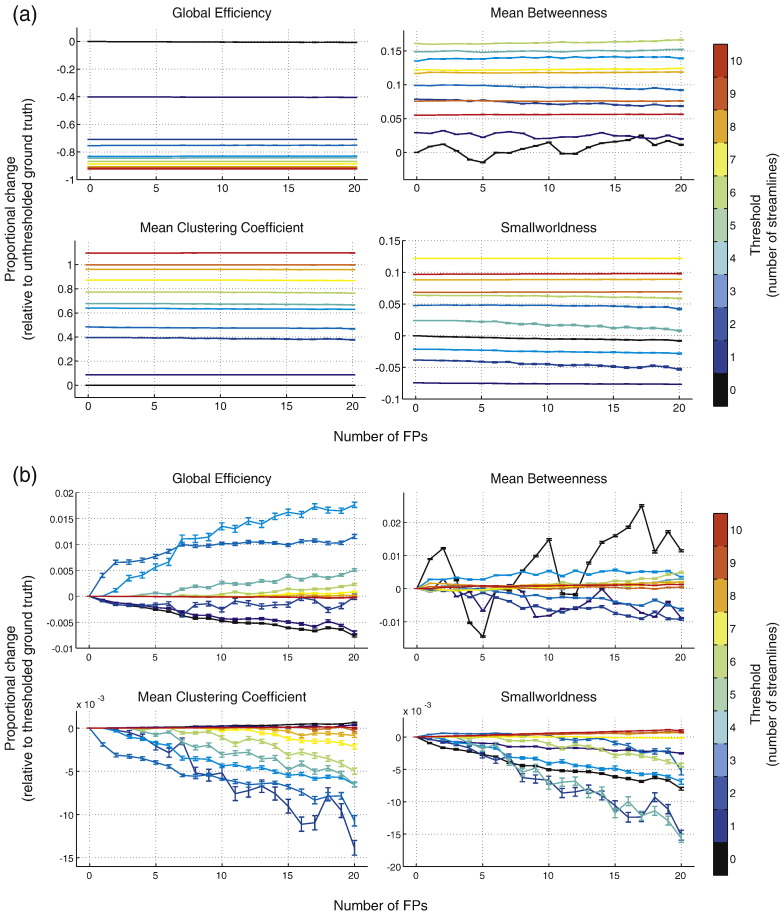
Effect of FPs when thresholds were applied. Thresholds are coded by colour with black indicating results for the unthresholded network. (a) Proportional change relative to the original unthresholded ground truth. (b) Proportional change relative to the thresholded ground truth network.

**Fig. 7:**
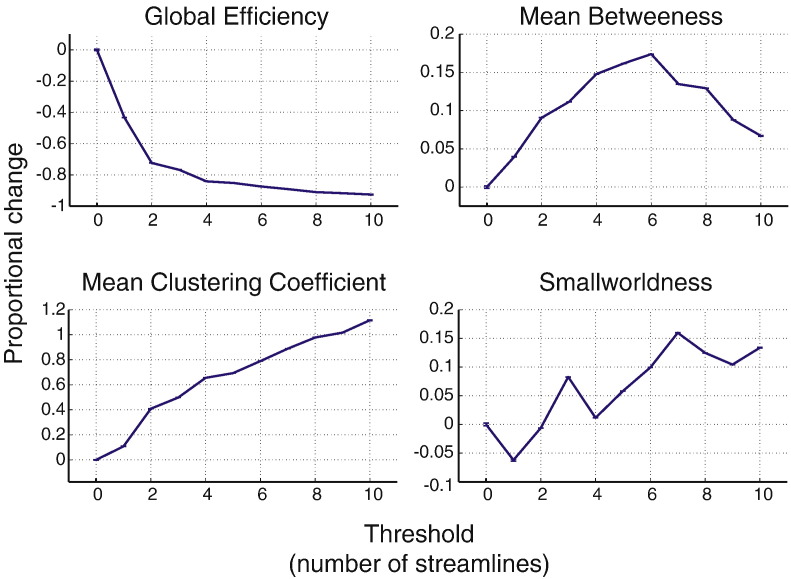
Overall effect of thresholding on network metrics, measured by proportional change. Error bars indicate standard error across iterations and FP counts.

Fig. 6a shows the proportional effect of thresholding relative to the unthresholded network while Fig. 6b shows the proportional change relative to the thresholded network. Thresholding has the effect of supressing the impact of FPs within-threshold. Network metrics of altered networks obtained at higher thresholds are in greater agreement with the metrics derived from the ground truth metrics, provided the same threshold is applied to that ground truth network. However, there is substantial bias across thresholds, which is shown in Fig. 7. All network metrics deviate significantly from the unthresholded ground truth network. The effects of thresholding differed across network metrics. Global efficiency increased with increasing thresholds while mean clustering coefficient decreased with increasing thresholds. Mean betweenness and smallworldness showed a non-monotonic pattern of change.

### Discussion

The first set of experiments investigated the effects of FPs on a model network and examined the effectiveness of thresholding to overcome the confounding effect of FPs. We show that FPs have a hugely detrimental effect on the quantification of network topology. We found differential effects of FP-NEs and FP-EEs, which makes the impact of FPs particularly difficult to predict, even if FP rates can be reliably estimated. However, applying a threshold to the corrupted network does effectively reduce the bias due to FPs.

Identifying the appropriate threshold will depend on the underlying FP rate, which is often unknown. Thresholding also introduces its own bias, which needs to be corrected. It is therefore difficult to make comparisons between network metrics directly. Statistical comparisons will be able to correct for this bias to an extent. However, given the unpredictable pattern of threshold bias in some network metrics, statistical comparisons are therefore likely to be unstable across thresholds.

In the following set of experiments, we examine how statistics for network metrics vary across thresholds in a large dataset. We also test the detectability of genuine group differences in the presence of this instability.

## Experiment 2a — effects of thresholding on statistical inference in random groups

This experiment examines the instability of statistical inference in networks where the FP rates are unknown. A statistically stable metric would be expected to vary smoothly across thresholds. In contrast, a statistical test whose results do not vary smoothly across thresholds will more likely be due to instability in the measurement, and lead to incorrect inferences being made.

### Method

All 248 tractography datasets from the ALSPAC cohort were used. HARDI data acquisition and parameters were the same as for experiment 1. However, no *a priori* constraints or pruning were used to exclude anatomically inconsistent streamlines, thus the number of FPs in each dataset was unknown.

The same four network metrics were measured as in the previous experiments. In addition, two non-network connectivity metrics were also computed as controls: the total number of edges and the number of streamlines across the whole computed network.

Subjects were randomly assigned to one of two groups of 124 subjects, in each of 250 permutations. Within each permutation, and for each network and non-network metric, Mann–Whitney *U*-tests were performed across a range of thresholds (0 < *τ* < 30 streamlines). This test was chosen because not all network metrics tested positively for normality consistently across thresholds (see Supplementary material S4). We quantified instability, by computing $\dot{\sigma }$; an analogue of the standard deviation that only includes deviations of the *U*-statistic between adjacent pairs of thresholds, as opposed to all pairs as computed by the traditional standard deviation.

(1) $${\dot{\sigma }}_{U}=\sqrt{\frac{\sum_{\bar{\tau }}^{n-1}\frac{1}{2}{\left(U_{\tau }-U_{\tau +1}\right)}^{2}}{n-1}}$$

This can equivalently be expressed as the L2 norm of the derivative of the statistics across thresholds.

(2) $${\dot{\sigma }}_{U}=\left\|\frac{dU}{d\tau }\right\|$$

where ***U*** and ***τ*** are the statistics and thresholds expressed as vectors.

We opted for this approach instead of computing the variance in the standard way because we wish to examine variability of the statistic only between adjacent thresholds. Although only noise is effectively measured in the test statistic, the apparent topology at particular thresholds will have strong dependency of those at adjacent thresholds. Therefore, it should be expected that the statistic varies smoothly from one threshold to another.

### Results

**Fig. 8:**
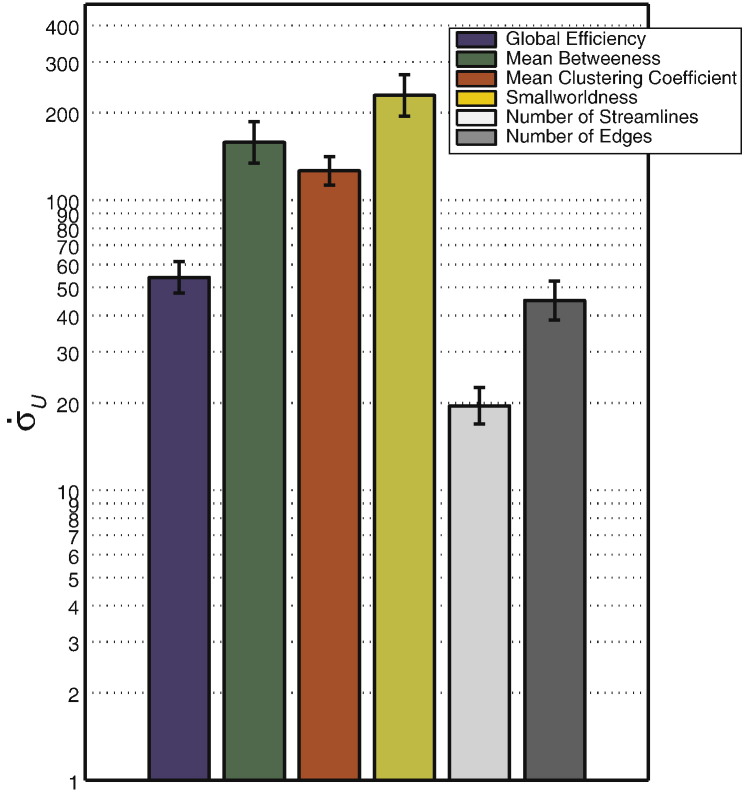
Results of experiment 2a. Stability measures (see main text) of *U*-tests performed on network and non-network control metrics across thresholds. Error bars show standard error across 100 permutation.

Results are shown in Fig. 8. All network metrics have significantly higher instability compared to the non-network metrics, with the exception of global efficiency, which has a level of instability similar to that of the number of edges. Smallworldness shows the highest instability while the number of streamlines shows the lowest instability.

## Experiment 2b — effects of thresholding on statistical inference with genuine group differences

In the previous experiment, statistical instability was measured in randomly assigned groups. Although our data show network metrics have more statistical instability compared to non-network metrics, this experiment still does not show how genuine group differences manifest in the presence of this instability. In the following experiment we investigated the effects of genuine group differences on statistical inference.

### Method

In the same 248 datasets, half the subjects were randomly assigned to a ‘healthy’ group and the other half to an ‘atrophied’ group. In the latter, the number of interhemispheric streamlines was reduced. The proportion of streamlines removed from interhemispheric edges was randomly sampled from a half-normal distribution with a mean of 0 and a standard deviation (denoted *ξ*) varying from 0 (i.e., no atrophy) to 0.3 in increments of 0.05. Atrophy of interhemispheric pathways have been implicated in several neurological and psychiatric conditions (Booth et al., 2011; Di Paola et al., 2010; Fields, 2008; Innocenti et al., 2003; Renard et al., 2014) and therefore is a realistic simulation of a clinical sample. This type of atrophy should also lead to changes in all network metrics tested.

Network metrics were computed for each network and thresholded at levels of 0 < *τ* < 30. Statistical comparisons were made with Mann–Whitney *U*-tests for each threshold. *U*-statistics were also computed for 1000 random permutations of the healthy-atrophied group assignments, to generate an estimate of the null-distribution of *U*-statistics. Two control metrics were also computed and tested: total number of edges and number of streamlines.

To additionally test that the effects were consistent and not due to underlying noise in the data, the healthy-atrophied group assignments were randomised prior to inducing atrophy. This was performed for 10 randomisations with atrophy applied at *ξ* = 0.25.

### Results

**Fig. 9:**
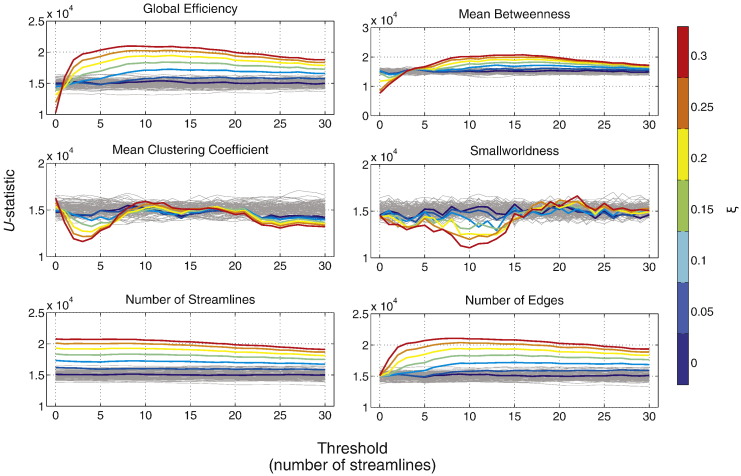
*U*-statistics obtained from comparing network and non-network metrics from healthy and atrophied groups. Each level of atrophy (*ξ*) is coded by colour. U-statistics for group permutations are shown in grey.

Results are shown in Fig. 9. Each of the network-metrics showed significant group effects across a range of thresholds. In all cases the magnitude of the apparent effect was proportional to *ξ*. The *U*-statistics for the global efficiency and mean betweenness produced broad peaks across a wide range of thresholds. *U* for global efficiency deviated from the null distribution at *τ* ≥ 1 and peaked at *τ* = 10. *U* for mean betweenness significantly deviated from the null distribution at *τ* ≥ 5 and peaked at *τ* = 14. For clustering coefficient significant effects were seen in narrower threshold ranges. *U* for mean clustering coefficient was significant for 1 ≤ *τ* ≤ 7 and peaks at *τ* = 3. *U* for smallworldness was significant at 7 ≤ *τ* ≤ 15 with a peak at *τ* = 10.

In contrast, the non-network metrics showed very consistent effects. The number of streamlines was consistently significant across all thresholds, while number of edges showed significant effects for *τ* ≥ 1, which is similar to the pattern seen for global efficiency.

**Fig. 10:**
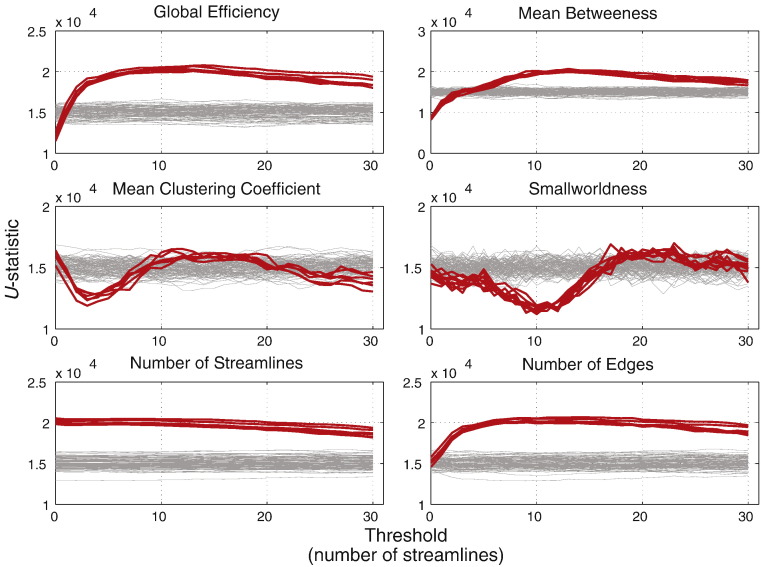
*U*-statistics obtained from comparing network metrics from healthy and atrophied groups with 10 reassignments of group prior to inducing atrophy (*ξ* = 0.25).

Fig. 10 shows the same comparisons made with different assignments of the healthy and atrophied groups (*ξ* = 0.25). The effects seen are consistent across randomisations although there is slightly more variability in *U* for mean clustering coefficient and smallworldness.

## Multi-threshold permutation correction (MTPC)

We have shown in experiment 2b that network metrics compared between groups with genuine topological differences remain statistically significant, despite the instabilities reported in experiment 2a. However, the thresholds at which the group differences appear statistically significant are not predictable. In some measures (e.g., global efficiency) group differences remain significant across a wide range of thresholds, while for others (e.g., mean clustering coefficient) the window is narrower. The threshold at which the peak effect is seen is also inconsistent between network metrics.

These results can be explained analytically as shown in Appendix A. Here, we showed that the parameters that determine the visibility of the group are dependent on the true connectome structure and the distribution of FPs, neither of which are known *a priori*. The range of thresholds in which a significant effect can be observed is therefore highly unpredictable. Therefore it is necessary to take a more data-driven, agnostic approach to searching for significant group effects.

To this end, we propose a new approach: multi-threshold permutation correction (MTPC), which performs statistical mapping on the graph metrics along thresholds and identifies clusters where a sustained group effects can be identified. The MTPC method can additionally tackle some of the standard issues in neuroimaging statistics such as the multiple comparisons problem and assumptions of the data distribution. There are three main components to the approach: (1) permutations (2) multiple thresholds and (3) cluster size.

(1). Permutation correction is well established as a way to identify significant clusters of voxels in standard statistical parametric mapping (SPM) analyses (Maris and Oostenveld, 2007; Nichols and Holmes, 2002; Smith and Nichols, 2009). This approach is particularly useful when the researcher has no *a priori* hypothesis as to the location of the effect of interest. Hence, it is suitable for detecting group effects with unknown windows of visibility, as is the case with graph metrics. This approach samples a null-distribution of test-statistics by permuting group assignments, and then comparing the original test statistic to this distribution. The maximum of the null test statistic across the whole image is usually sampled as a means of correcting for multiple comparisons. Given the complex and non-linear operations involved in network analysis, permutation tests are generally more advisable than standard parametric tests, since they do not make assumptions about the distributions of these measures (Hosseini et al., 2012; Langer et al., 2013; Simpson et al., 2013; Zalesky et al., 2010).
(2). As demonstrated in the previous experiment, the effects of FPs can be corrected by applying thresholds. As it is unknown what the underlying FP rate is, a wide range of thresholds is applied and the effects under each threshold are quantified.
(3). Given that reliable statistics should vary smoothly across thresholds, a rapidly varying result is less likely to be due to a genuine group effect. To further ensure that any super-critical clusters are not due to random variation in the test statistic, the size of each super-critical cluster is computed from the super-critical area under the curve (AUC). This can then be compared to the expected size of clusters due to noise. This can be easily derived from the mean AUC of the super-critical clusters for the permuted statistics.

### The MTPC pipeline

**Fig. 11:**
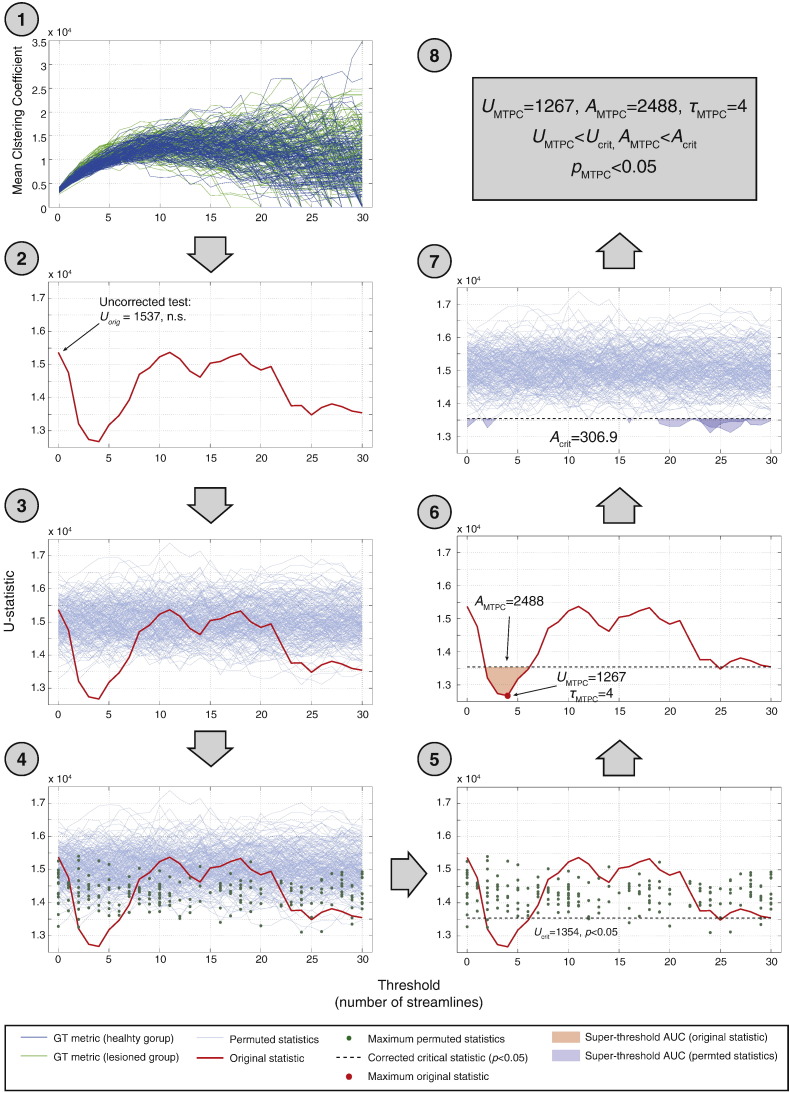
Flowchart of the MTPC pipeline, demonstrated on comparisons of clustering coefficient between the healthy and atrophied groups in experiment 2b (*ξ* = 0.2). Steps of the pipeline are explained in the text. (1) Network metrics are computed for each subject and threshold, up to *m* = 30 streamlines. (2) *U*-Statistics were computed between groups for each threshold. The initial uncorrected unthresholded test yields a non-significant result. (3) Groups were permuted *n* = 1000 times and -statistics computed for each permutation. (4) The maximum statistic across thresholds was taken for each permutation, generating null distribution of *U*-statistics (note we use the term maximum with respect to the direction of the observed effect). (5) The upper 95th percentile of this distribution is taken as the critical value for *U* which is *U*_crit_ = 1354. (6). One super-critical cluster was identified with a peak of *U*_MTPC_ = 1267 at *τ* = 4 with a super-critical AUC of *A*_MTP*C*_ = 2488. (7). The mean super-critical AUC was *A*_crit_ = 306.9. (8) Both *U*_MTPC_ and *A*_MTPC_ exceed their respective critical values. Therefore, the null hypothesis is rejected.

The MTPC pipeline for network metrics is as follows (also see Fig. 11 for a graphical representation):

1. Apply thresholds, *τ*, to the networks, from 0 to *n_τ_* and compute network metrics for all networks across all thresholds.
2. Compute test statistics $S_{H}$ (e.g., *t*, *F* or *U*) on network metrics with the correct group assignments for each threshold.
3. Permute the group assignments across *n*_rand_ iterations and recompute test statistics for each permutation and each threshold, giving a distribution of null test statistics at each threshold.
4. Take the maximum test statistic across all thresholds for each permutation, resulting in one summarised null statistic for each permutation. To additionally correct for multiple comparisons of node-level metrics across regions, take the maximum across nodes as well as thresholds.
5. Identify the critical value (denoted here as $S_{\mathrm{crit}}$) for the test-statistic from the top *α*th percentile of the null test statistics, where *α* is the desired confidence level (e.g., 5%).
6. Identify clusters where the true test statistic is higher than the critical value for each threshold and compute the AUC for these clusters (denoted *A*_MTPC_). The peak statistic and the corresponding threshold are denoted S_MTPC_ and *τ*_MTPC_, respectively.
7. Compute a critical AUC from the mean of the super-critical AUCs for the permuted tests (denoted *A*_crit_).
8. Reject the null hypothesis if the AUC of the significant clusters exceeds the critical AUC (*A*_MTPC_ > *A*_crit_).

### Parameter selection

Two parameters for MTPC, *n*_rand_ and *n_τ_* need to be specified. There are no definitive ways for choosing these parameters but some recommendations are described below:

1. Number of permutations (*n*_rand_). Ideally the set of all possible permutations should be computed for a complete test. However, for even modest sample sizes this is often unfeasible. Generally, the higher the number of permutations, the greater the precision of the corrected test. The limiting factor is often computational resources and time. A general guideline is that 500–1000 permutations are acceptable for an initial test. If any statistics are found to be close to the critical value, 10,000–100,000 permutations are recommended for a more robust test (Edgington and Onghena, 2007; Jöckel, 1986).
2. Range of thresholds (*n_τ_*). An advantage of MTPC is its agnostic approach to thresholding. *a priori* assumptions need not be made. Instead, a wide range of thresholds should be tested. As with *n*_rand_, the larger *n_τ_* is, the more robust the test. Because the MTPC performs statistical tests on each threshold separately, there will not be a dilution of effect with wider threshold ranges (unlike other multi-threshold approaches such as the AUC method). However, to ensure an efficient use of computational resources, some strategies may be employed to ensure the best candidate range is chosen. Some studies have used the mean node degree as a criterion for thresholding networks (Meunier et al., 2009; van den Heuvel et al., 2009). This is useful with other non-discrete connectivity measures (e.g., coherence) with different scales. Another example is Bassett et al. (2006) who used a minimum node degree of 2ln(*N*) as an upper limit for their chosen threshold, of the assumption that the core ‘skeleton’ of the network is retained above this level, beyond which the networks are implausible. Another data-driven approach for limiting the range of thresholds is to choose a maximum threshold limit, which retains a particular architectural characteristic, such as a small-world or rich-club architecture (van den Heuvel and Sporns, 2011).

The workflow of this procedure is presented in Fig. 11 using the example of mean clustering coefficient from experiment 2b (*ξ* = 0.2). The parameters of the MTPC were *n*_rand_ = 1000 and *n_τ_* = 30. In this case, the critical *U*-statistic from the computed null distribution at *α* = 0.05 is *U_crit_* = 1354. A cluster at 2 ≤ *τ* ≤ 6, exceeds *U_crit_*, with a peak of *U_MTPCx_* = 1267 at *τ*_MTPC_ = 5. The super-critical AUC of this cluster is *A_MTPC_* = 2488, which exceeds the critical cluster size of *A_crit_* = 306.9. The group difference in mean clustering coefficient is therefore deemed to be significant. This contrasts with the standard uncorrected test, which would deem this effect to be non-significant (*U_orig_* = 1537, n.s.).

## Experiment 2c — applying MTPC to the atrophied-healthy group comparison

In this section we take the data from experiment 2b and apply the MTPC method to test its ability to detect group effects across varying levels of atrophy.

We compare the method to the more established approach of computing AUCs for network metrics prior to computing group statistics. This approach is an attractive data-driven way of consolidating effects across thresholds and is less computationally intensive than MTPC. However, by summing across thresholds at the subject-level, the inter-threshold variability in the test statistics, as demonstrated in experiment 2a, will be lost in the summary measures. This is potentially problematic as this variability may be important for robust estimating the null distribution. Also, as mentioned previously, the AUC method is likely to dilute genuine group effects if they only manifest in narrow ranges of thresholds.

### Method

The MTPC method was applied to the data from experiment 2b. *U*-Statistics were computed comparing the healthy and atrophied group at each level of atrophy (0 ≤ *ξ* ≤ 0.3) and thresholds (0 ≤ *τ* ≤ 30). *U*-Statistics were additionally computed for n = 1000 group permutations. The peak *U*-statistic (*U*_MTPC_) and the super-critical AUC (*A*_MTPC_) were recorded.

For the AUC method, the AUC of each network metric across thresholds was computed for each subject. *U*-statistics were then computed for the group comparison of the AUCs (denoted *U*_AUC_) and the same set of permutations performed for MTPC. The test statistic was then tested for significance against the null distribution.

The critical values for both methods were computed for *p* < 0.05. The values of *U*_AUC_, *U*_MTPC_ and *A*_MTPC_ and their respective critical values were plotted against *ξ*. The sensitivity of each statistic was measured using the minimum detectable atrophy, derived from the value of *ξ* at the intercept between the relevant statistic and its respective critical value.

### Results

**Fig. 12:**
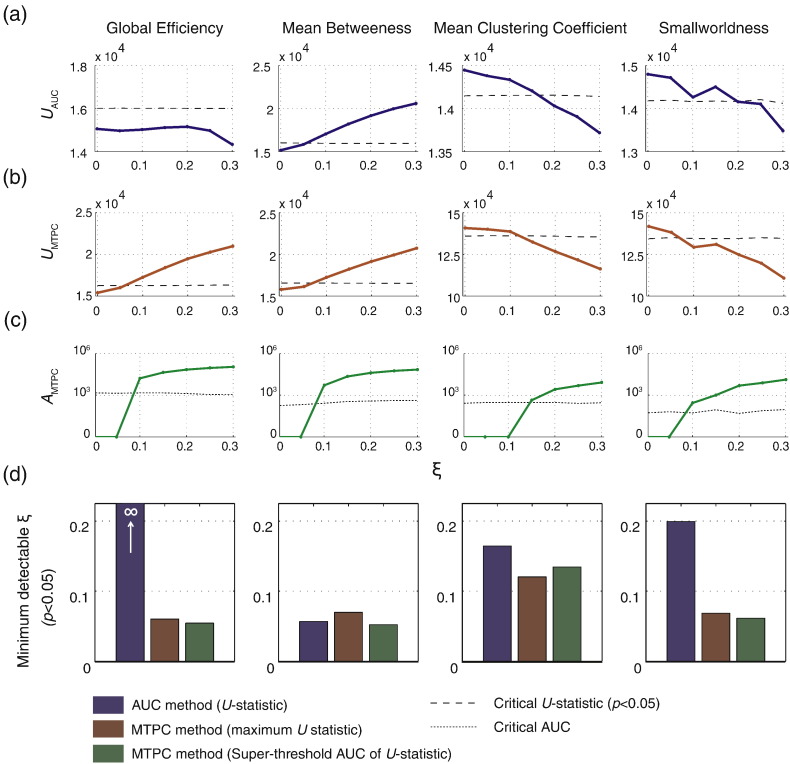
Results of the AUC and MTPC methods comparing network metrics from healthy and atrophied groups from experiment 2b across levels of atrophy (*ξ*). Dotted lines indicated the respective critical values. (a) *U*-statistics (*U*_AUC_) obtained from comparisons of AUC of network metrics computed at subject-level. (b). Maximum *U*-statistic (*U*_MTPC_) computed using the MTPC method (c) Super-critical AUCs of *U*-statistics (*A*_MTPC_) using the MTPC method. (d). The minimum detectable *ξ* for each method, obtained from the intercept of the relevant statistic with its respective critical value.

Fig. 12 shows the resultant statistics for the two methods for each network metric. The AUC method failed to identify an effect of global efficiency for any *ξ*. In contrast, the MTPC method was able to identify an effect at *ξ* ≈ 0.1. For mean betweenness, both AUC and MTPC methods detected the effect at *ξ* ≈ 0.1. For mean clustering coefficient, The MTPC method showed a slightly higher sensitivity (*ξ* ≈ 0.1) compared to the AUC method (*ξ* ≈ 0.15). For smallworldness, the MTPC method again showed higher sensitivity (*ξ* ≈ 0.1) compared to the AUC method (*ξ* ≈ 0.2).

## Experiment 3 — application of the MTPC method

Here, the MTPC method was applied to structural networks obtained from adults suffering traumatic brain injury (TBI) and healthy controls. Network analysis of this dataset has previously been published in (Caeyenberghs et al., 2014).

### Method

Diffusion MRI data were acquired from 37 adult TBI patients and 27 healthy controls. Full details of the participant inclusion criteria, MRI acquisition, pre-processing and derivation of the connectivity matrices are provided in (Caeyenberghs et al., 2014). Edges were weighted by the proportion of streamlines relative to the total number of streamlines.

The same four network metrics used in the previous experiments were computed. Group effects were tested with independent-samples *t*-tests. For all four metrics, the *t*-statistic was computed at thresholds from 0 ≤ *τ* ≤ 50. Permuted *t*-statistics were computed across *n* = 1000 iterations. MTPC was applied with a significance threshold of *α* = 0.05.

### Results

**Fig. 13:**
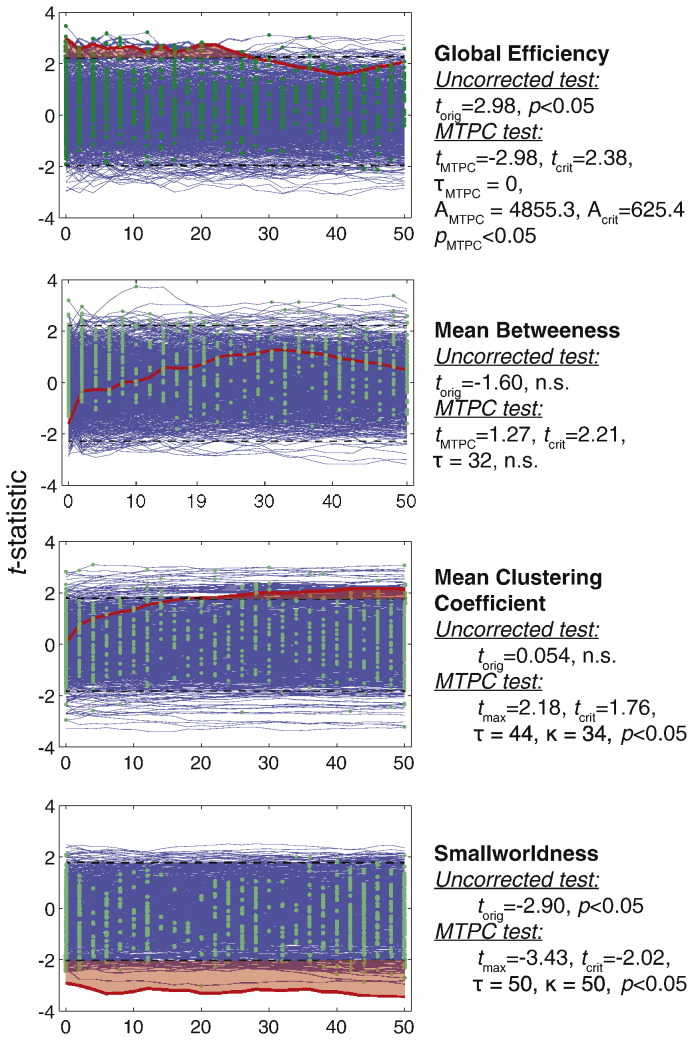
MTPC applied to the statistical tests using the data of (Caeyenberghs et al., 2014).

The permuted and unpermuted *t*-statistics are plotted in Fig. 13, with the corresponding results of MTPC. Two metrics show a significant group effect: Global efficiency and smallworldness. Global efficiency showed a super-critical cluster at 0 ≤ *τ* ≤ 26, which exceeded the critical cluster size (*t*_MTPC_ = − 2.98, *t*_crit_ = − 2.38, *A*_MTPC_ = 476.8, *A*_crit_ = 191.9, *τ*_MTPC_ = 0, *p*_MTPC_ < 0.05). Smallworldness showed a consistent super-critical effect across all thresholds, which tested significant with MTPC *t*_MTPC_ = − 3.43, *t*_crit_ − 2.02, *A*_MTPC_ = 2985.3, *A*_crit_ = 240.5, *p*_MTPC_ < 0.05, *τ*_MTPC_ = 50). The other two metrics were found to be non significant. Mean betweenness did not produce any super-critical clusters, and therefore tested as non-significant (t_MTPC_ = 1.27, t_crit_ = 2.21, n.s.). Mean clustering coefficient did produce a super-critical effect at *τ* ≥ 20. However the size of this cluster did not exceed the critical cluster size and therefore tested non-significant with MTPC (*t*_MTPC_ = 2.18, *t*_crit_ = 1.76, *A*_MTPC_ = 364.1, *A*_crit_ = 760.3, n.s.).

## Discussion

Graph theory (GT) is a powerful framework for quantifying topological properties of brain networks. However, the sensitivity to false positives (FPs) in the reconstructed networks can give rise to spurious inferences. This study investigated these effects in four commonly used network metrics: Global efficiency, mean clustering coefficient, mean betweenness and smallworldness. We then investigated the effects of thresholding on these metrics and the stability of statistical comparisons with these metrics. We developed a new method of multi-threshold permutation correction (MTPC) that reliably detects genuine group differences in network metrics.

### Effects of false positives

The results of our first experiment show that even a single false positive will have significant impact on inferred network metrics. This is true for cases where FPs created new edges (FP-NEs) or FPs added to existing edges (FP-EEs), causing an overestimation of the edge weights. The only exception is mean clustering coefficient, which only deviated significantly at 6 or more FP-NEs. More importantly, the direction of bias produced by FPs varied depending on where the FPs lie. Global efficiency showed a strong positive bias in the presence of false edges, but a slight negative bias to the presence of additional streamlines in existing edges. This difference is due to the normalisation of the edge weights to the number of streamlines. While the inferred efficiency of the affected edges increases, the weights of the unaffected edges will be scaled down causing an overall decrease in efficiency. The addition of edges causes a much stronger bias in global efficiency, and therefore the streamline normalisation produces no noticeable effect. Clustering coefficient showed a comparatively weak, but still significant effect. Again FP-NEs show a negative effect while FP-EEs lead to a positive effect. A network with a high degree of clustering will appear less clustered when artefactual edges are added, but if existing edges are enhanced without additional edges, the apparent strength of the existing clusters will be enhanced. Smallworldness shows a consistent negative effect in both FP-NEs and FP-EEs. Betweenness centrality showed monotonic change due to FPs, particularly for FP-EEs. Previous studies have found, that measurements of centrality are particularly unstable (Borgatti et al., 2006; Costenbader and Valente, 2003; Segarra and Ribeiro, 2014).

Importantly, the normalisation of edge weights means that the observed effects are not only due to changes of network density. The FP-EE experiment also shows significant changes without changes in the number of edges. An additional analysis (experiment 1b) also shows that alterations of network topology, without changes in the number of edges or edge weights can still result in significant differential changes in network metrics. Although we corrected for network density in our study, this may not always be appropriate. If reduction in density is a primary driving force in a topological change, then controlling for density may result in failure to detect the effect of interest. This is likely to be the case in studies of neurodegenerative conditions. However, if there is a hypothesised network reorganisation, not due to change in density, which is more likely for psychiatric or neurodevelopmental conditions, then controlling for density is appropriate. The decision whether or not to control for density should depend on the specific nature of the hypothesised effect.

There is substantial variability in the regional sensitivity to FPs across network metrics, with global efficiency and smallworldness showing the greatest regional variability (see Supplementary material S3). The most heavily implicated region was the posterior and middle cingulate cortex, and precuneus. This is interesting in the context of previous studies that have analysed general topological features of structural brain networks. A number of studies in healthy adults (Gong et al., 2009; Hagmann et al., 2008; van den Heuvel and Sporns, 2011) and clinical populations (Tijms et al., 2013; van den Heuvel et al., 2010) have identified the posterior cingulate as a critical hub in structural brain networks. This suggests that FPs in particularly critical parts of the network introduces greater detrimental impact on inferred global topology.

### Effects of thresholding

Thresholding has been shown to significantly reduce the effects of FPs. When comparing network metrics across FP rates *within* thresholds, the rate of FPs has less effect as threshold increases. This means that at low thresholds, a small number of FPs can be effectively controlled for, while higher thresholds are required to correct higher FP rates.

Although *within* thresholds, there is a reduced effect of FPs, when comparing network metrics *across* thresholds, it becomes clear that thresholding comes at the expense of introducing its own bias, which for some network metric is unpredictable (particularly for mean betweenness). For some metrics, this bias follows a predictable pattern, for example, global efficiency shows an exponential decrease with increasing thresholds. It may be possible to model the effects of thresholding on specific network metrics, but such an approach requires *a priori* assumptions of the FP rate in the inferred network. In Supplementary Materials S1, we show the same is true for node-level network metrics, with the effects of thresholding showing very large variability across regions as well as thresholds. This bias will again lead to erroneous estimates of network metrics.

We also showed, in experiment 2a, that network metrics are significantly more unstable than non-network measurements of the same networks. FPs and thresholds will both bias the topology of the network (sometimes in opposing directions) such that their effects on network metrics will be difficult to predict across thresholds. However, the results from experiment 2b showed that genuine group differences can still be detected in network metrics and that the magnitude of the observed effects are roughly proportional to the size of the true group effect (see Fig. 12). Global efficiency and mean betweenness showed strong and consistent effects at thresholds of *τ* ≥ 3. Smallworldness and mean clustering coefficient also showed effects, but spanning a more limited range of thresholds. This is due to the effects of rapid phase transitions in the connectome structure with increasing thresholds, contributing to fluctuating variance (see Appendix A). Furthermore, the ranges in which the effects are seen differ. For clustering coefficient, this is approximately 1 ≤ *τ* ≤ 7, and smallworldness, 5 ≤ *τ* ≤ 15. This means that, although results show detectable group effects for all network metrics, the effects are not necessarily seen in the same threshold ranges. A multi-threshold approach is therefore still required. There may also be the possibility of scenarios where threshold bias can counteract the visibility of group effects, leading to false acceptance of a null hypothesis.

### Factors affecting bias due to FPs and thresholding

Consideration should be given to the type of network change that is expected between groups. In our simulated example, we induced topological changes by simulating changes in the corpus callosum pathways, which has been implicated in several neurological and psychiatric conditions (Booth et al., 2011; Di Paola et al., 2010; Fields, 2008; Innocenti et al., 2003; Renard et al., 2014). However, other types of atrophy may result in altered sensitivity to thresholding. For example, distributed loss of connections is more likely to impact on clustering coefficient and global efficiency than more focal insults to the network. In addition, the measurement used for weighting the graph edges requires consideration. Some weighting strategies will influence how thresholds affect derivation of network metrics (Hagmann et al., 2008; Sporns et al., 2005). Further investigation into the stability of measurements using other connectivity indices is required. Here we have only shown effects using streamline-based indices, but there are a wide range of measures which can also be used to weight structural networks, such as fractional anisotropy, myelination or FOD-based indices (Dell'Acqua et al., 2013). Differences in these weighting measurements may manifest in the threshold-induced bias and statistical instability differently to the weighting measures used here. Another factor that is likely to be important is the consistency of these measurements across sessions or subjects. For this reason it is unlikely that network metrics from different modalities (e.g., comparing structural and functional networks), or even using different acquisition and analysis parameters, are comparable with each other. For example, tractography performed with an angular threshold of 45° would have fewer FPs than with a threshold of 60°. The hidden differences in FP rates will lead to variation in their sensitivity to thresholding. As such, comparisons of network metrics between networks inferred using different processing pipelines, parameters or different modalities should not be made without a good estimate of the FP rates of each network and making appropriate correction for these differences.

FP rates may be managed by imposing anatomical *a priori* constraints on tractography immediately after tract reconstruction. Constraints such as pruning the streamlines when they enter grey matter, as performed in this study, will achieve this to an extent. Manual segmentation of all existing fibre pathways is usually unfeasible. However, there are automated clustering techniques that can perform clustering of whole-brain connectomes with *a priori* anatomical information. For example, using a coregistered white-matter atlas to constrain the tractography output (Maddah et al., 2005), defining streamlines as unique chains of ROIs (Q. Wang et al., 2013) or using machine learning to capture salient features of tracts from a training set of exemplar tracts (Parker et al., 2013b). Unfortunately, even the most robust clustering methods will still be subject to some error. Given the low tolerance of network analysis to FPs, thresholding will still be a requirement. However, use of such methods will allow more confidence of a low FP rate and therefore safely reduce the maximum threshold to test.

Another issue to consider is the effects of false negatives (FNs). The MTPC method does not account for the effect of FNs and thresholding the network will inevitably lead to increases in the FN rate. For this reason it may be appropriate to derive the connectivity matrix using more lenient parameters to ensure the inferred networks have higher FP rates (which are easier to control for) than FN rates.

### Relevance to functional connectivity

Although our investigations focused on tractography-derived connectivity, the same issues highlighted here will also affect functional connectivity measures. Our findings are therefore relevant to network analysis of functional networks and similar biases should be expected.

In EEG/MEG functional connectivity, a common source of FPs is artefactual correlations arising due to volume conduction or magnetic field spread (Nolte et al., 2004; Nunez et al., 1997). Additionally, in the source domain, the ill-posed nature of the source inverse problem leads to additional short-range cross talk between reconstructed sources, causing more FP correlations (Hui et al., 2010; Sekihara et al., 2011). While measures have been developed which are more robust to these effects, such as imaginary coherency (Nolte et al., 2004) and phase lag index (Stam et al., 2007), all these still suffer from the problem of higher-order artefacts, which are much more difficult to control (Drakesmith et al., 2013). Also, there is lack of sensitivity to deep sources, which will result in subcortical parts of the functional neural network being permanently invisible, meaning that network metrics will only ever capture features of a sub-set of the true network.

In fMRI-derived functional connectivity (e.g., cross-correlations), aberrant connections can arise from assumptions made about the uniformity of the haemodynamic response to neural activity. Haemodynamic response functions vary considerably in their time-course across different regions. This can lead to highly erroneous connections to manifest. Physiological noise that has not been effectively controlled, such as respiration and heart rate is highly correlated with the BOLD signal, further contributing to spurious correlations (Murphy et al., 2013). One study examining the stability of fMRI-derived network metrics as a function of acquisition time found network metrics stabilise at sufficiently long acquisition times (Whitlow et al., 2011). However, this finding does not discount any systemic bias due to correlated noise sources.

### Advantages of MTPC

The results of experiment 2b show genuine group effects are visible even with the high instability of statistics across thresholds. Our proposed MTPC approach is developed to detect such effects in a robust manner.

MTPC addresses a number of issues in the statistical analysis of network metrics. Firstly, the method obtains a true null distribution of test statistic by permutation. This is a valuable approach for network metrics given the highly complex and non-linear operations involved in their computation (Hosseini et al., 2012; Langer et al., 2013; Simpson et al., 2013). The permutation framework also allows for the correction of multiple comparisons across thresholds and, in the case of node-level metrics, across regions. Second is the issue of arbitrariness of the selection of a single threshold. Although some have proposed measures to more accurately estimate the correct threshold to use (e.g., Bassett et al., 2006), our approach systematically computes null distributions across a feasible range of thresholds, and tests each point for significant effects. This minimises the *a priori* assumptions required for choosing a single threshold. Third, the method accounts for instability in statistics by testing for effects across thresholds and imposing the condition that any clusters of apparent significant effects should exceed the expected cluster size due to noise.

**Fig. 14:**
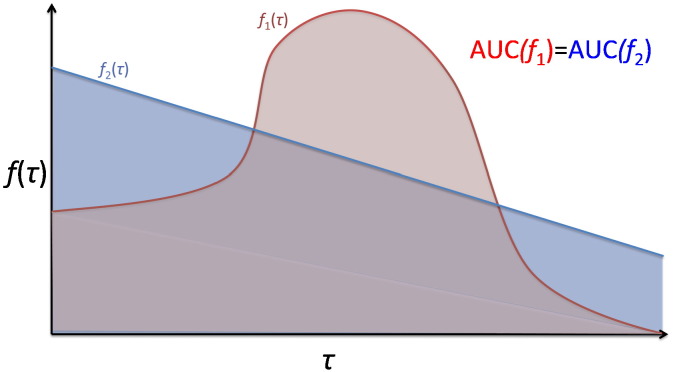
A toy illustration of a scenario where the AUC method will fail to identify a difference between graph metrics with very different profiles along τ. The two networks (red and blue) show distinct behaviour across τ, yet computing the AUC will reveal no differences between the two networks.

In experiment 2c, we show that the MTPC approach is more sensitive to group effects than the existing AUC method. This approach also considers network metrics at multiple thresholds (Cao et al., 2013; Hosseini et al., 2012; Rubinov et al., 2009; Zhang et al., 2011). However, in contrast to MTPC, network metrics are summarised across thresholds at the subject-level prior to group comparisons. As a result, the AUC method is less sensitive to effects traversing narrow ranges of thresholds, as the values at non-significant thresholds will dilute the observed effect. For example, if one subject shows a consistently high but sub-critical measurement, this can have a higher AUC than a subject whose measurement is low through most thresholds but significantly high in a narrow window of thresholds, as demonstrated in experiment 2b. A similar confound can take place in the opposite direction. See Ginestet et al. (2011) for a more detailed discussion of this problem (see also Fig. 14).

The improved performance of MTPC can also be attributed to the ability to consider variance of test statistics across thresholds, and therefore allowing a more accurate estimate of the null distribution. This is suggested by the fact that the two measurements showing effects in only narrow thresholds (mean clustering coefficient and smallworldness) are two measurements MTPC outperforms the AUC method on.

A more critical observation of the AUC method is the total failure to detect any effect in global efficiency, despite the relatively stable effects. This is most likely due to the sharp exponential decay of global efficiency with increasing thresholds (see Fig. 7). The computed AUCs will be heavily biased towards the values at small thresholds, where the group effects do not manifest (see Fig. 9). Therefore, effects at higher thresholds will be less detectable. The MTPC method does not suffer this problem, as each threshold is tested separately.

### Considerations for MTPC

An issue with MTPC is the assumption that topological differences between groups do not lead to different sensitivities to the varying threshold. For example, one group might show a larger change in clustering coefficient in the step from one threshold to another. Andreotti et al. (2014) have found that differences in network density between groups can inflate group differences and suggest normalising for network density. Therefore, thresholding such that the density of the networks is matched at each threshold rather than a uniform scale on the connectivity measure, should be more robust. This matched strategy approach has been utilised in some network analysis studies (Bonilha et al., 2012; Dennis et al., 2011; Schindler et al., 2008). However, as discussed above, the appropriateness of correcting for density will depend on the specific hypotheses being tested.

Another potential problem with MTPC approach is the assumption of equivalence of statistics performed at different thresholds across different network metrics. In the results of experiment 2b, there is some variation in the location of the most significant peak. Particularly, for mean clustering coefficient (peak effect at *τ* = 3) and smallworldness (peak effect at *τ* = 10). If one were to search independently for effects in these three measures using MTPC, we would identify effects in some network metrics that are not necessarily apparent at the same thresholds as other network metrics (e.g., mean clustering coefficient will not be deemed significant thresholded at *τ* = 10, where the most significant peak lies for smallworldness). It is unclear whether the inferred effects at these peaks can be treated as equivalent measures of the same network. The network metrics derived at different thresholds are, strictly, measuring different networks, but are being treated as the same network. It may therefore be more appropriate to constrain the search across thresholds such that only network metrics with peaks in the same windows are treated as significant. However, if there are large differences in the location for significant peaks, this will lead to more ambiguity as to whether a significant effect exists overall. In addition, our results show there is large variation in how thresholds affect different network metrics. Van Wijk et al. (2010) illustrate flaws with assuming that network metrics between groups will be equivalent and that the appropriate threshold will itself be depended on the expected network metrics. It is therefore more prudent to search a range of thresholds for each metric independently to accommodate for this variability.

### Conclusion

Network metrics are highly susceptible to FPs, but applying thresholds to the networks prior to network analysis can reduce these effects. Unfortunately this introduces additional bias to the network metrics and it is not possible, without *a priori* knowledge, to predict this bias, which is rarely known in neuroimaging experiments. There is also high instability in statistical inference in these metrics. However, genuine group effects can be seen in some network metrics, across a range of thresholds. The range of thresholds across which the effect can be detected is not known, but by performing permutation tests across a wider range of thresholds, it is possible to identify clusters of significant group effects to correctly identify genuine group effects. The MTPC approach is more sensitive to group effects than the existing AUC approach. This approach will more robustly identify genuine group differences in network metrics and can be used for testing network metrics derived from both structural and functional connectivity. The approach of graph theory, and particularly statistics in graph theory, is still in its infancy in neuroimaging, with little consensus or standardization of analyses. The presented approach is therefore an important step beyond those currently implemented in the literature.

## Figures and Tables

**Fig. 15:**
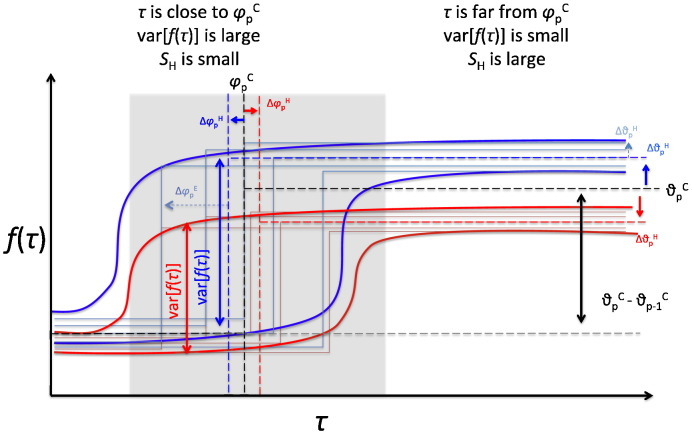
A toy illustration of the fluctuations of variance. Two groups of networks (red and blue) with different topologies are shown around a core transition point, *φ*_p_^C^ with subject-specific and group specific offsets Δ*φ*_p_^E^ and Δ*φ*_p_^H^, respectively. Bold lines indicate the variance of *f*(τ) for each group. The variance of *f*(τ) is inflated around *φ*_p_^C^ leading to low $S_{H}$. As τ moves away from *φ*_p_^C^, variance reduces and $S_{H}$ is larger, allowing the group differences to be identified.

## Appendix A

Here we present an analytical explanation for narrow windows of thresholds in which statistically significant effects can be observed. We show for a sample of graphs with a hypothesised group effect, significant group effects are only detectable in small windows of thresholds, which, without knowledge of the true underlying topology and the distribution of FPs, cannot be determined.

Given a set of graphs, where each member, G_*k*,*l*_ is a graph for subject *l* in group *k*. Each group has *n_k_* graphs and there are *m* groups in total.

(1) $$G=\left\{\left\{G_{1,1},G_{1,2}\ldots G_{1,n_{1}}\right\},\left\{G_{2,1},G_{2,1}\ldots G_{2,n_{2}}\right\}\ldots \left\{\ldots G_{k,l}\ldots \right\}\ldots \left\{G_{m,1},G_{m,2}\ldots \;G_{m,n_{m}}\right\}\right\}\text{.}$$

Each graph in G is represented by a set of *n*_V_ vertices, *n*_E_ edges and *n*_E_ edge weights.

(2) $$G_{k,l}=G\left(V,E,W\right)$$

We use the *n*_E_ × *n*_E_ weighted connectivity matrix **W**_*k*,*l*_ as a proxy for G_*k*,*l*_ where *w*_*i*,*j*_ are the weights corresponding to the edges $E_{V_{i}↔V_{j}}$. Each graph represented by W_*k*,*l*_, can be expressed as a sum of matrices

(3) $$W_{k,l}=C+{\Delta H}_{k}+{\Delta E}_{l}$$

where **C** is the core structure of the connectome common to all subjects and groups, Δ**H**_*k*_ is the change in **C** due to effects specific to group *k*. Δ**H**_*k*_ can include group differences that influence any topological measurement (e.g., global efficiency, clustering coefficient, betweenness centrality, etc.). Δ**E**_*l*_ is the change in **C** due subject-specific factors. This can include true individual differences in topology as well as false positive edge weights arising from the data acquisition or analysis. We will only consider graphs with positive edge weights, so **C** has only positive elements. Δ**H**_*k*_ and Δ**E**_*l*_ can have positive or negative elements but none such that any element in **W**_*k*,*l*_ is negative.

To quantify some topological property of **W**_*k*,*l*_ we use some graph metric function, denoted *f*(**W**_*k*,*l*_). The goal of the analysis is to compute a statistic testing whether or not the hypothesized topological effects of Δ**H**_*k*_ on *f*(**W**_*k*,*l*_) exists. $S_{H}\left(G\right)>S_{\alpha }$ indicates a significant effect, where $S_{\alpha }$ is the critical value of the test statistic at the acceptable type I error rate (e.g., *α* = 0.05).

We additionally assume that the variance (both subject-specific and group specific) of connectome topology in human brains is very small in the context of all possible variations of **C**.

(4) $$\forall i,j:\left|c_{i,j}\right|>\mathrm{var}\left[\Delta h_{i,j,k}\right]\wedge \left|c_{i,j}\right|>\mathrm{var}\left[\Delta e_{i,j,l}\right]$$

where *c*_*i*,*j*_ Δ*h*_*i*,*j*,*k*_ and Δ*e*_*i*,*j*,*l*_ are the *i*th and *j*th elements of **C**, Δ**H**_*k*_ and Δ**E**_*l*_, respectively.

To apply a threshold, we make W_*k*,*l*_ a function of threshold *τ*.

(5) $$W_{k,l}\left(\tau \right)=W_{k,l}\left(0\right)-T_{k,l}\left(\tau \right)$$

where, **W**_*k*,*l*_(0) denotes the unthresholded matrix and **T**_*k*,*l*_(*τ*) is the matrix of edge weights to be removed at of threshold *τ*, which is monotonically increasing, with elements *t*_*i*,*j*_ such that

(6) $$t_{i,j}=\left\{\begin{array}{ccc} \begin{array}{l} w_{i,j}\\ 0 \end{array} & \begin{array}{l} :\\ : \end{array} & \begin{array}{l} \tau <u_{i,j}\\ \tau \geq u_{i,j} \end{array} \end{array}\right)$$

where *u*_*i*,*j*_ are the elements of the matrix, **U**_*k*,*l*_, some criteria for thresholding. **U**_*k*,*l*_ may be the same as **W**_*k*,*l*_ or may be derived from some other measure (e.g., density, node degree, and cost-functions). We further denote $G\left(\tau \right)$ as the full set of graphs thresholded at *τ*.

For simplicity, we will assume throughout that **U**_*k*,*l*_ = **W**_*k*,*l*_. However, where **U**_*k*,*l*_ ≠ **W**_*k*,*l*_, the relationship between components of **W**_*k*,*l*_ expressed in Eq. (4) should instead apply to the equivalent components in **U**_*k*,*l*_. The components of **W**_*k*,*l*_ need not obey this relationship in this case.

We can show simply that there is dependency of $S_{H}\left(G\left(\tau \right)\right)$ on *τ*. Consider a set of graphs $\hat{G}$, equivalent to G where Δ**E**_*l*_ = 0. i.e., all variability in $\hat{G}$ is due only to the effect in ΔH_*k*_. In this case, $S_{H}\left(G\right)>S_{\alpha }$ will always be true, as long as |Δ**H**_*k*_| > 0. When thresholds are applied $\hat{G}\left(\tau \right)\text{,}$ all effects observed are due to Δ**H**_*k*_ and there is no between-subject variability. Therefore $S_{H}\left(\hat{G}\left(\tau \right)\right)$ is constant across all *τ*. Under this condition, the AUC approach (see introduction) would be valid for detecting group effect (see Ginestet et al., 2011). However, outside of this unique case, there will always be some variation of $S_{H}\left(G\left(\tau \right)\right)$. If this variation is sufficiently large, the AUC approach will fail to identify genuine group effects. Ginestet et al. (2011) illustrates this in more detail (see also Fig. 14).

Below we illustrate two ways in which the sensitivity of $S_{H}\left(G\left(\tau \right)\right)$ to Δ**H**_*k*_ is highly variable. We first describe a broad interval along τ where effects are detectable, and secondly, show how the statistic fluctuates over thresholds.

### Interval of detectable group effects

We can express *f*(*τ*) in terms of it constituting components in a similar way to Eq. (3):

(7) $$f\left(W_{k,l}\left(\tau \right)\right)=f^{C}+\Delta f_{k}^{H}+\Delta f_{k,l}^{E}\left(\tau \right)$$

where

(8) $$\begin{array}{c} f^{C}=f\left(C\right)\\ \Delta f_{k}^{H}=f\left(C+{\Delta H}_{k}\right)-f^{C}\\ \Delta f_{k,l}^{E}\left(\tau \right)=f\left(W_{k,l}\left(\tau \right)\right)-f^{C}-\Delta f_{k}^{H} \end{array}$$

i.e., *f*^C^ is the topology of the core connectome, **C**. Δ*f*_*k*_^H^ is the group-specific change in *f*(**W**_*k*,*l*_(*τ*)) and Δ*f*_*k*,*l*_^E^(*τ*) is the subject-specific change in *f*(**W**_*k*,*l*_(*τ*)), which includes the error due to **T**_*k*,*l*_(*τ*). Note that only the subject-specific effects vary over τ.

The sensitivity of $S_{H}\left(G\left(\tau \right)\right)$, will depend on the ratio of the inter-group variance to the inter-subject variance.

(9) $$S_{H}\left(G\left(\tau \right)\right)\propto \frac{var\left[\Delta f_{k}^{H}\right]}{var\left[\Delta f_{k,l}^{E}\left(\tau \right)\right]}\text{.}$$

If, as is common in network analysis, we assume that the elements of ΔE_*l*_ are small compared to the non-zero elements Δ**H**_*k*_, then var[Δ*f*_*k*,*l*_^E^(*τ*)] will decrease most rapidly at lower τ. However, when τ is sufficiently larger, elements of ΔH_*k*_ will be removed by **T**_*k*,*l*_(*τ*), resulting a large increase in var[Δ*f*_*k*,*l*_^E^(*τ*)]. We can therefore define approximate limits along τ where $S_{H}\left(G\left(\tau \right)\right)\;>S_{\alpha }$ is most likely as [*τ*_*a*_, *τ*_*b*_] such that:

(10) $$\begin{array}{c} \tau_{a}<\tau_{b},\mathrm{where}\\ \tau_{a}=\underset{\tau }{\mathrm{argmax}}-\frac{d}{d\tau }var\left[\Delta f_{k,l}^{E}\left(\tau \right)\right]\\ \tau_{b}=\underset{\tau }{\mathrm{argmax}}\frac{d}{d\tau }var\left[\Delta f_{k,l}^{E}\left(\tau \right)\right] \end{array}$$

The interval [*τ*_*a*_ , *τ*_*b*_], will be dependent on the structure of **C** and Δ**E**_*l*_ which are not known *a priori*.

### Fluctuating variance of graph metrics

Even if the assumption made in Eq. (10) is valid, this is not sufficient to describe the fluctuations in variance of graph metrics. Fluctuations arise due to the interaction of subject-specific variance with the core connectome topology (and to a lesser extent, the group-specific topology). The topology of graphs typically show large phase transitions when their edges are manipulated, such as by thresholding (Janson et al., 2011). The phase transitions can be either positive or negative; therefore *f*(*τ*) is not necessarily monotonic. We create a simple model to approximate such behaviour using a stepwise function.

(11) $$f\left(W_{k,l}\left(\tau \right)\right):=f\left(\tau \right)=\sum_{p=1}^{n_{\varphi }-1}\vartheta_{p}\left\{\begin{array}{c} 1:\tau \in \left[\varphi_{p},\varphi_{p+1}\right]\\ 0:\tau \notin \left[\varphi_{p},\varphi_{p+1}\right] \end{array}\right)$$

where *f*(*τ*) is the graph metric being modelled as a function of *τ*. *φ*_*p*_ is the location on *τ* where phase transition *p* takes place, *ϑ*_*i*_ is the value of *f*(*τ*) between two phase transitions [*φ*_*p*_, *φ*_*p* + 1_] and *n*_*φ*_ is the total number of phase transitions that take place across τ. The position and size of the phase transitions depends on the specific topology of **W**_*k*,*l*_(*τ*).

We express the parameters of the modelled version of *f*(*τ*) in a similar way as in Eq. (7), such that:

(12) $$\varphi_{p}=\varphi_{p}^{C}+\Delta \varphi_{p}^{H}+\Delta \varphi_{p}^{E}$$

where *φ*_*p*_^C^ is the locations on *τ* were phase transition *p* take place due to the changes in the core connectome structure **C**, Δ*φ*_*p*_^H^ is the group-specific offset in *φ*_*p*_ and Δ*φ*_*p*_^E^ is subject-specific offset in *φ*_*p*_. The same principle also applies to *ϑ*_*p*_.

(13) $$\vartheta_{p}=\vartheta_{p}^{C}+\Delta \vartheta_{p}^{H}+\Delta \vartheta_{p}^{E}$$

Note that from here on, we consider the effects of Δ*φ*_*p*_^E^ on *ϑ*_*p*_^C^ and the effects Δ*φ*_*p*_^E^ of Δ*φ*_*p*_^H^ as equivalent (with the exception of a caveat mentioned below). Therefore, only the effects of Δ*φ*_*p*_^E^ on *φ*_*p*_^C^ are demonstrated.

Following Eq. (4), we assume the greatest variation in *φ*_*p*_ and *ϑ*_*p*_ is that due to **C** while the smallest variation is due to ΔE_*l*_.

(14) $$var\left[\varphi_{p}^{C}\right]>var\left[\Delta \varphi_{p}^{E}\right]$$

and

(15) $$var\left[\vartheta_{p}^{C}\right]>var\left[\Delta \vartheta_{p}^{E}\right]$$

As *τ* approaches a particular core phase transition *φ*_*p*_^C^ with a variable offset Δ*φ*_*p*_^E^, the variance of *f*(*τ*) around these values will increase, approaching the variance of the magnitude of the phase transitions (the inter-transitional variance).

(16) $$\lim_{\tau \to \varphi_{p}^{C}}var\left[f\left(\tau \right)\right]\;=var\left[\vartheta_{p}-\vartheta_{p+1}\right]$$

Conversely, as τ approaches the intervals *between* phase transitions, $\frac{1}{2}\left(\varphi_{p}^{C}+\varphi_{p+1}^{C}\right)$, the variance Δ*f*_*k*,*l*_^E^(*τ*) will decrease, approaching the intra-transitional variance:

(17) $$\lim_{\tau \to \frac{1}{2}\left(\varphi_{p}^{C}+\varphi_{p+1}^{C}\right)}var\left[f\left(\tau \right)\right]\;=var\left[\vartheta_{p}\right]$$

The minimum possible intra-transitional variance only consists of variance due to Δ*ϑ*_*p*_^E^ while the maximum possible inter-transitional variance will consist of variance due to Δ*ϑ*_*p*_^E^ and *ϑ*_*p*_^C^. Therefore, these limits can be expressed as:

(18) $$\begin{array}{c} var\left[\vartheta_{p}\right]=var\left[\Delta \vartheta_{p}^{E}\right]\\ var\left[\vartheta_{p}-\vartheta_{p+1}\right]=var\left[\vartheta_{p}^{C}+\Delta \vartheta_{p}^{E}\right] \end{array}$$

And therefore, following Eq. (15), the variance at the inter-transitional point is always higher than that of the intra-transitional point.

(19) $$var\left[\vartheta_{p}-\vartheta_{p+1}\right]>var\left[\vartheta_{p}\right]\text{.}$$

And equivalently,

(20) $$var\left[f\left(\varphi_{p}^{C}\right)\right]\;>var\left[f\left(\frac{1}{2}\left(\varphi_{p}^{C}+\varphi_{p+1}^{C}\right)\right)\right]\;\text{.}$$

As a result, the subject-specific variance of *f*(*τ*) fluctuates between these limits, depending on the proximity of *τ* to the phase transitions *p*. Since Δ*f*_*k*_^H^ and *f*^C^ are invariant to τ (Eq. (8)), var[*f*(*τ*)] is proportional to var[Δ*f*_*k*,*l*_^E^(*τ*)]. Therefore, fluctuations in var[*f*(*τ*)] are inversely proportional to the fluctuations in $S_{H}\left(G\left(\tau \right)\right)$ (Eq. (9)). A toy illustration of this effect is shown in Fig. 15.

The rate at which var[*f*(*τ*)] varies, will depend on var[*φ*_*p*_^E^]. If var[Δ*φ*_*p*_^E^] = 0, there is no increase in variance except at the precise point the phase-transitions takes place. The graph metric variance will remain stable across the transition interval, i.e., var[*f*(*τ*)] = var[*ϑ*_*p*_]. As var[Δ*φ*_*p*_^E^] increases, the window of stable variance within each interval decreases. A large var[Δ*φ*_*p*_^E^], or a large *n*_*φ*_ will result in very narrow windows of stability, leading to increased var[*f*(*τ*)] and therefore poor sensitivity of $S_{H}\left(G\left(\tau \right)\right)$ to ΔH_*k*_.

The same principle applies to the effects of Δ*φ*_*p*_^E^ on group specific phase transitions, Δ*φ*_*p*_^H^, except that the increase in var[*f*(*τ*)] around Δ*φ*_*p*_^H^ will be group-specific. This will have a less pronounced impact as the overall subject-specific variance of *f*(*τ*) across all groups will be lower around Δ*φ*_*p*_^H^ than *φ*_*p*_^C^. However, this can also introduce heteroscedasticity to *f*(*τ*) which, depending on the choice of statistic, can bias the statistical inference.

In conclusion, var[*f*(*τ*)], and hence $S_{H}\left(G\left(\tau \right)\right)$, fluctuates considerably across τ. the parameters that determine the exact behaviour of var[*f*(*τ*)] are dependent on the structure of **C** and Δ**E**_*l*_, which are not known *a priori*. This necessitates a more data-driven agnostic approach to searching for intervals of τ where $S_{H}\left(G\left(\tau \right)\right)\;>S_{\alpha }$.

## Appendix B Supplementary data

Effects of FPs and thresholds on node-level GT metrics.

Comparison of proportional changes in GT metric with studies of clinical groups.

Edge-specific effects of FPs and thresholds.

Lillefors tests for normality.
