# Supplementary material for: Overcoming the effects of false positives and threshold bias in graph theoretical analyses of neuroimaging data
Source: Neuroimage. 2015 Sep;118:313–33. doi: 10.1016/j.neuroimage.2015.05.011 (PMC4558463; doi:10.1016/j.neuroimage.2015.05.011)
Supplement: Supplementary file 1 — Effects of FPs and thresholds on node-level GT metrics. [file mmc1.pdf]

Supplementary material S1: Effects of FPs and thresholds on node-level GT metrics

In addition to the four network-level GT metrics investigated in the main text, four node-level metrics were also investigated. These were clustering coefficient, betweenness, strength and modularity.
The results for experiments 1a and 1b for these metrics in each node are shown below (to minimise size of the plots, only results for right-hemisphere nodes are shown).

Figures S1.1-S1.4 show effects of FP-NEs.
Figures S1.5-S1.8 show effects of FP-EEs.
Figures S1.9-S1.12 show effects of thresholds.

As with the results for network-level metrics, FP-NEs and FP-EEs cause significant deviation from the ground truth with a small number of FPs in most regions. The trajectory of the effect varies between regions, with some regions showing negative effects, while others show positive effects. Modularity values are particularly variable.
Regional GT metrics show very high regional variability in the effect of thresholds. The trajectories also do not follow predictable patterns across thresholds

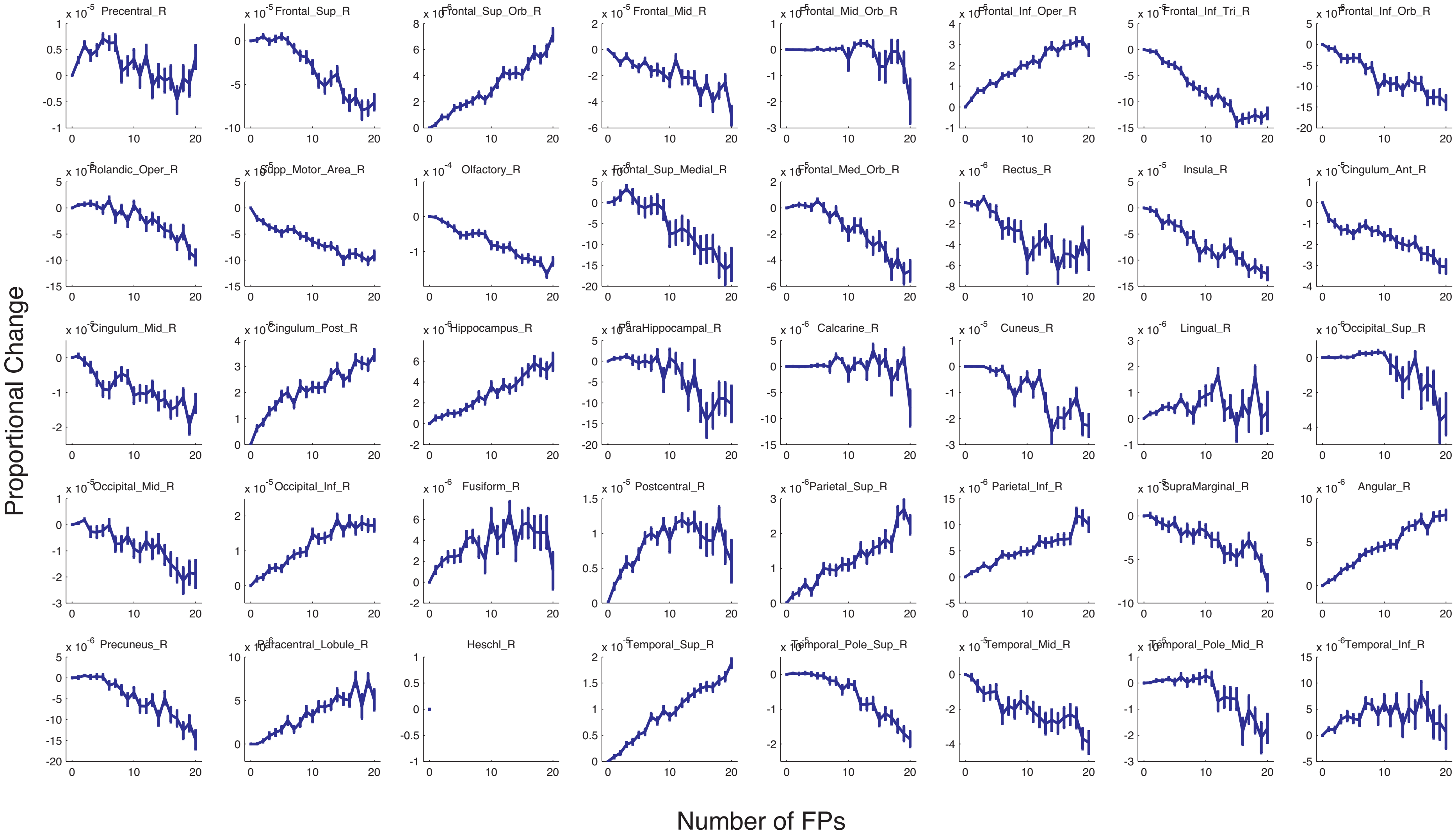

Fig. S1.1. Effects of FP-NEs on local clustering coefficient.

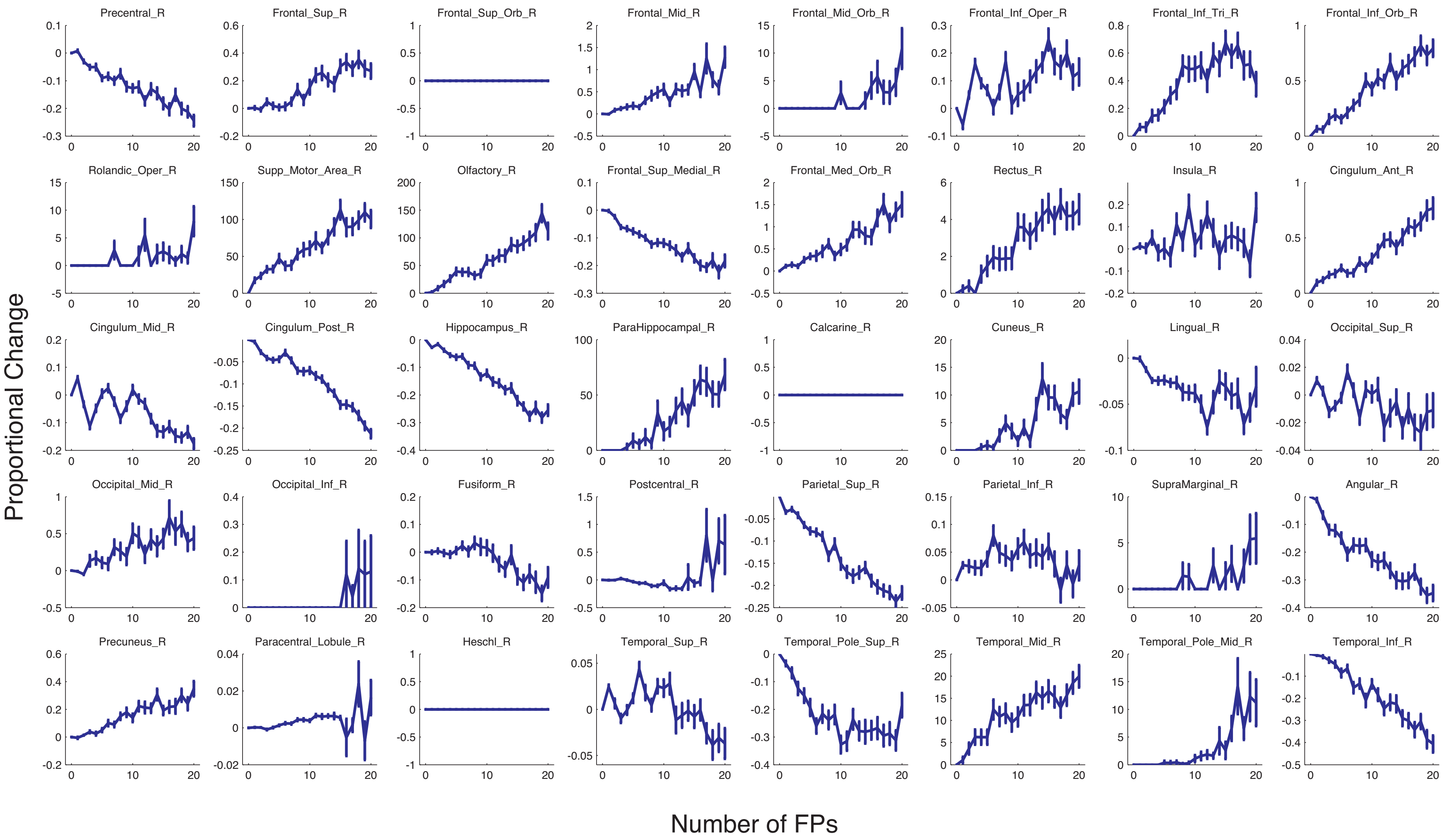

Fig. S1.2. Effects of FP-NEs on local betweenness.

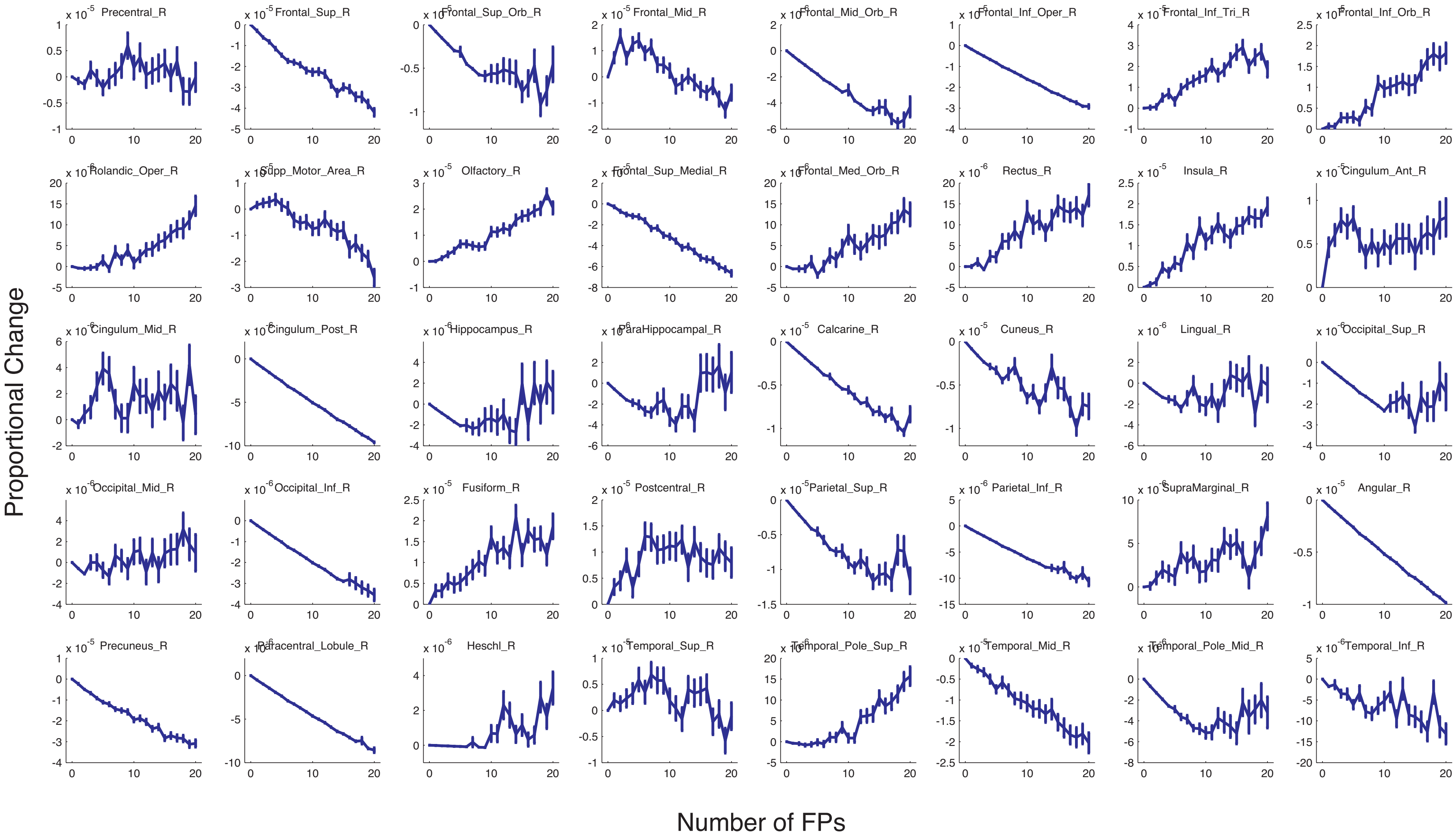

Fig. S1.3. Effects of FP-NEs on node strength.

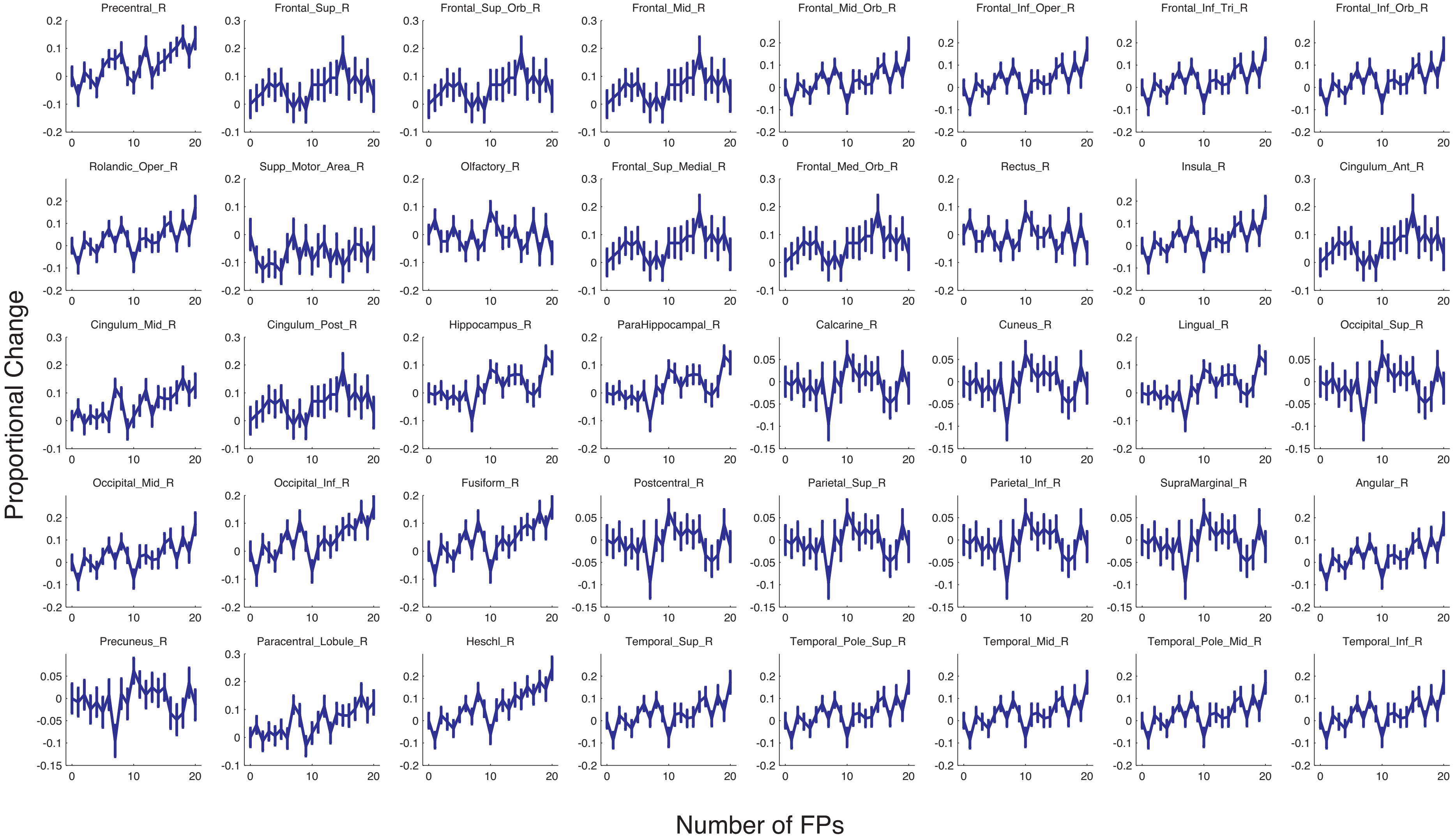

Fig. S1.4. Effects of FP-NEs on modularity.

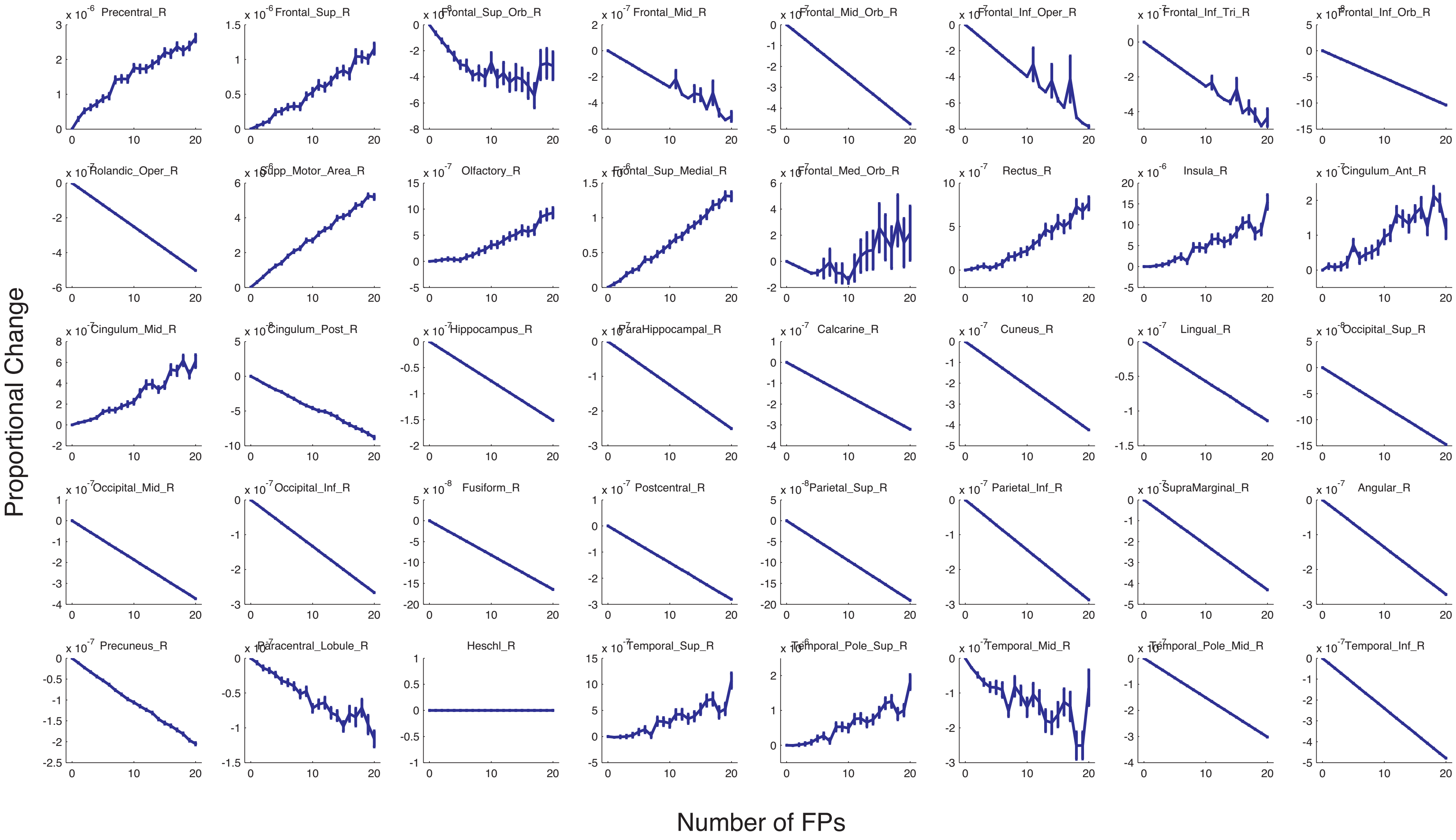

Fig. S1.5. Effects of FP-EEs on local clustering coefficient.

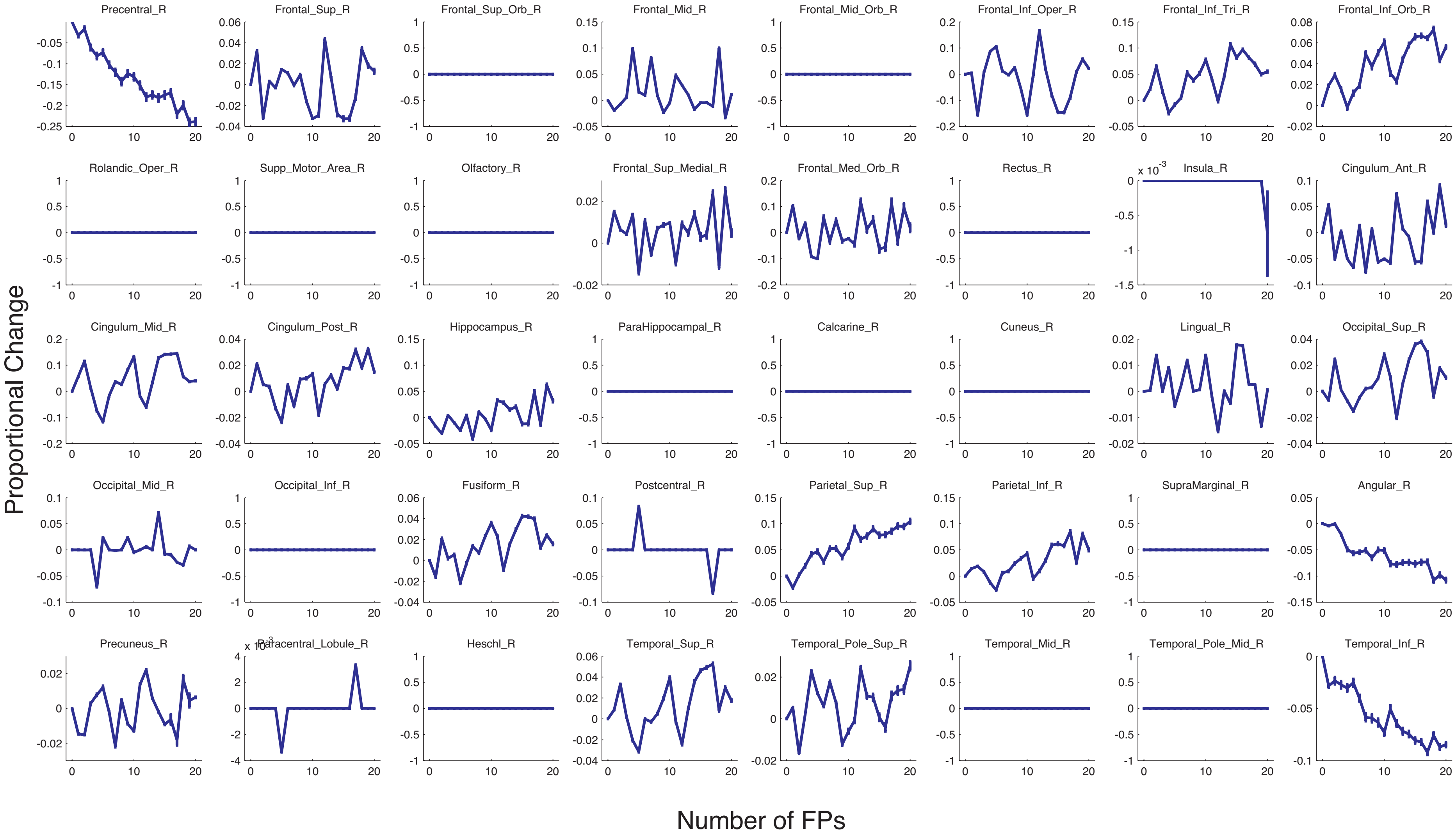

Fig. S1.6. Effects of FP-EEs on local betweenness.

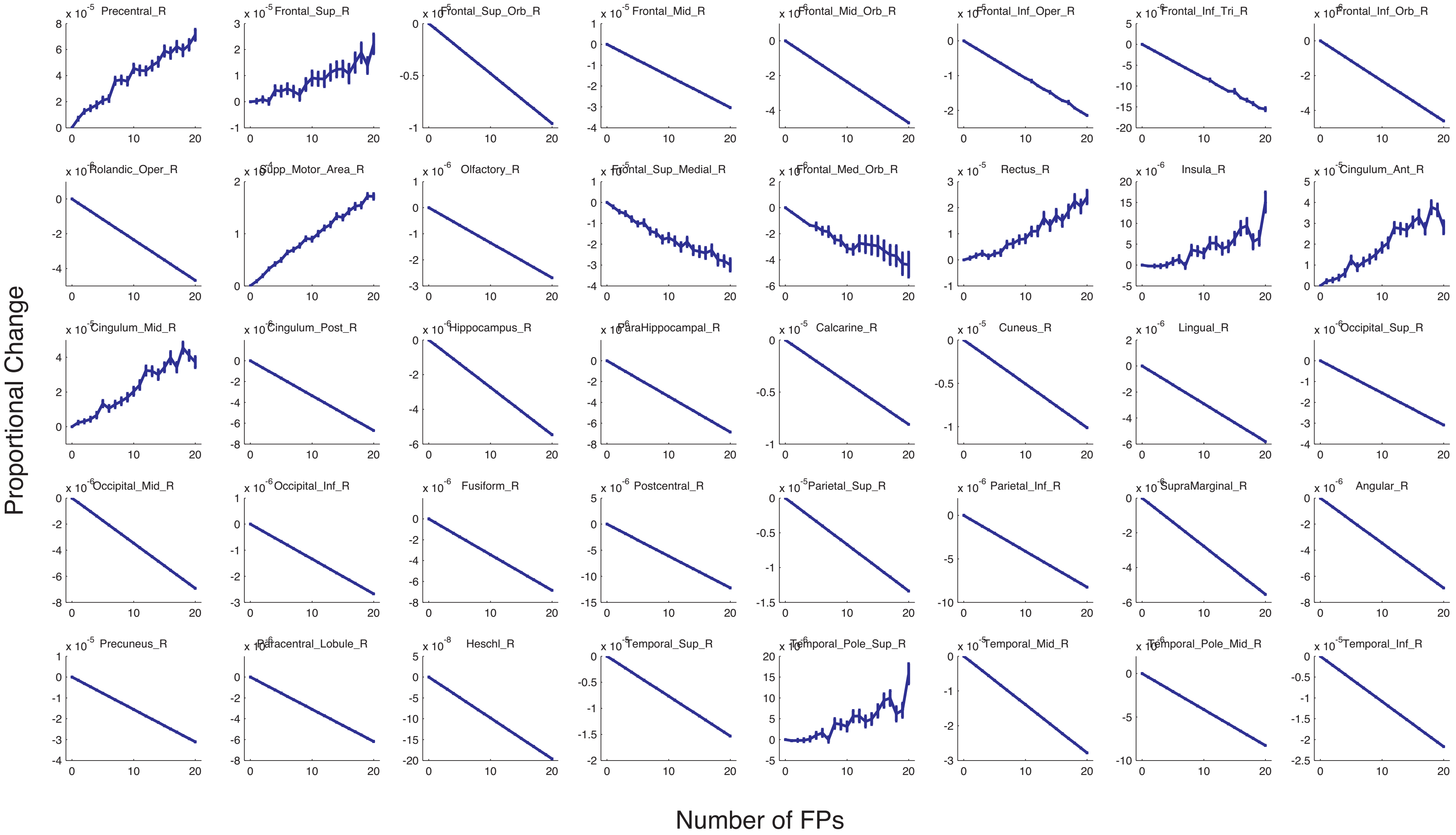

Fig. S1.7. Effects of FP-EEs on node strength.

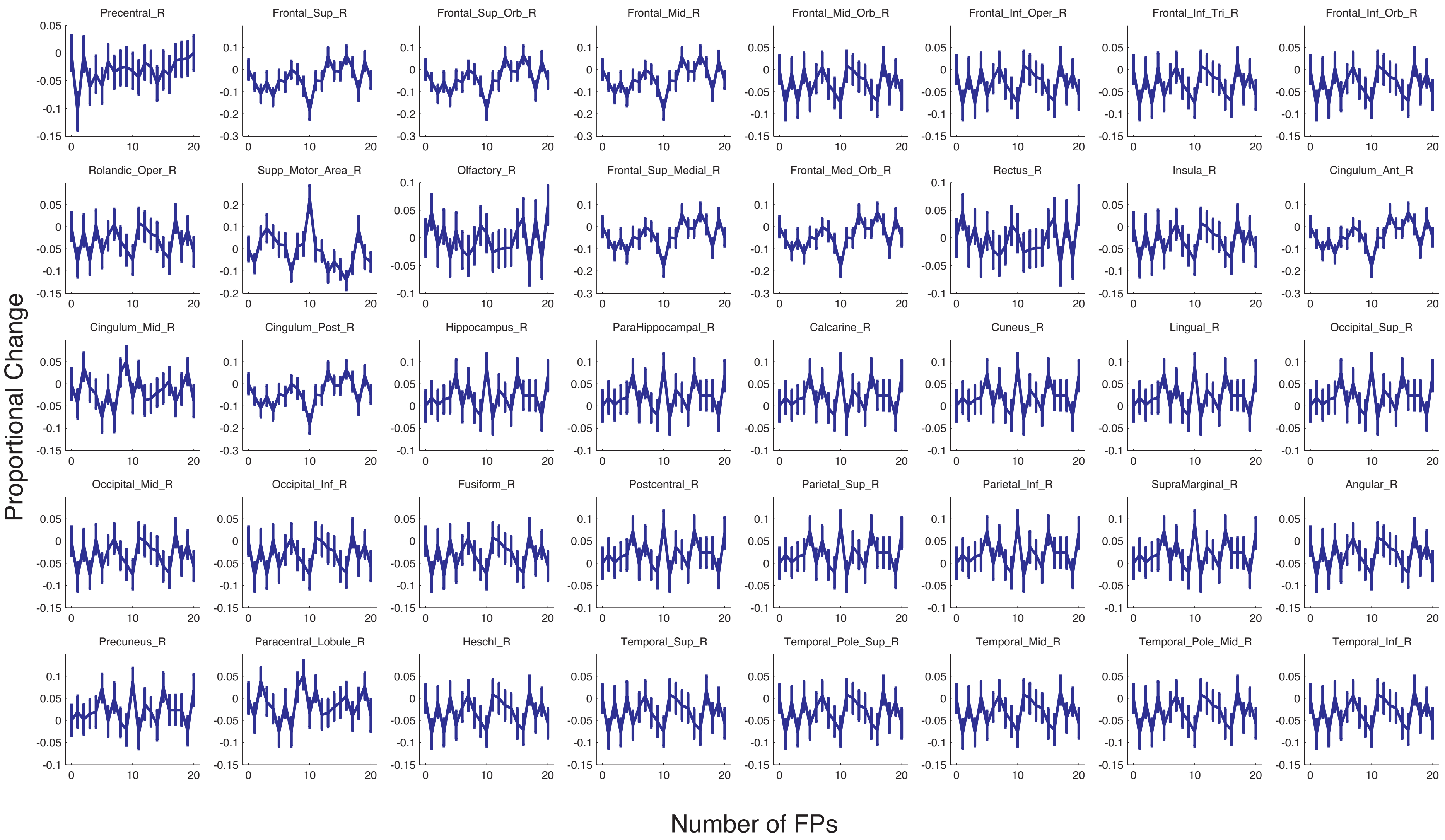

Fig. S1.8. Effects of FP-EEs rate on modularity.

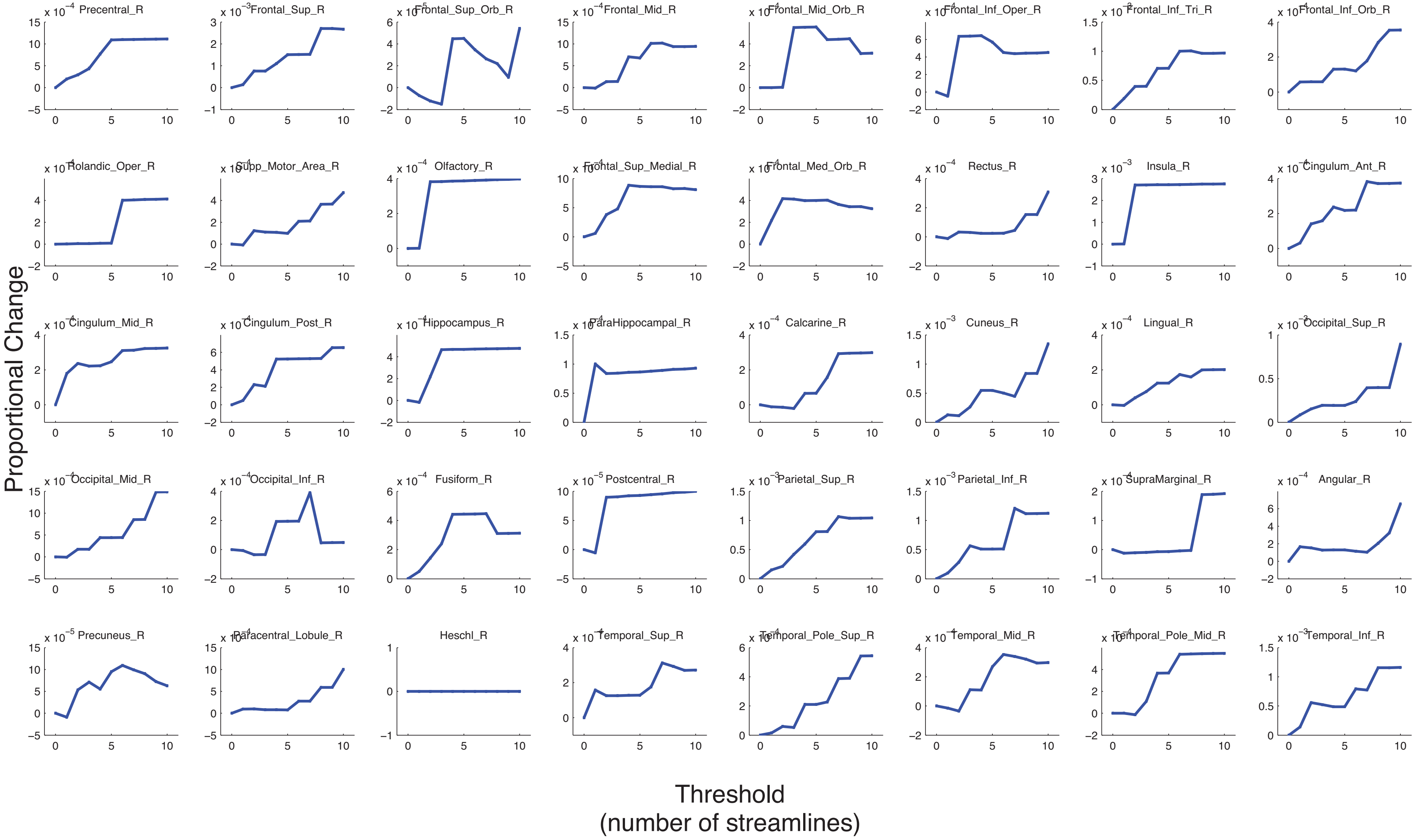

Fig. S1.9. Effects of thresholds on local clustering coefficient.

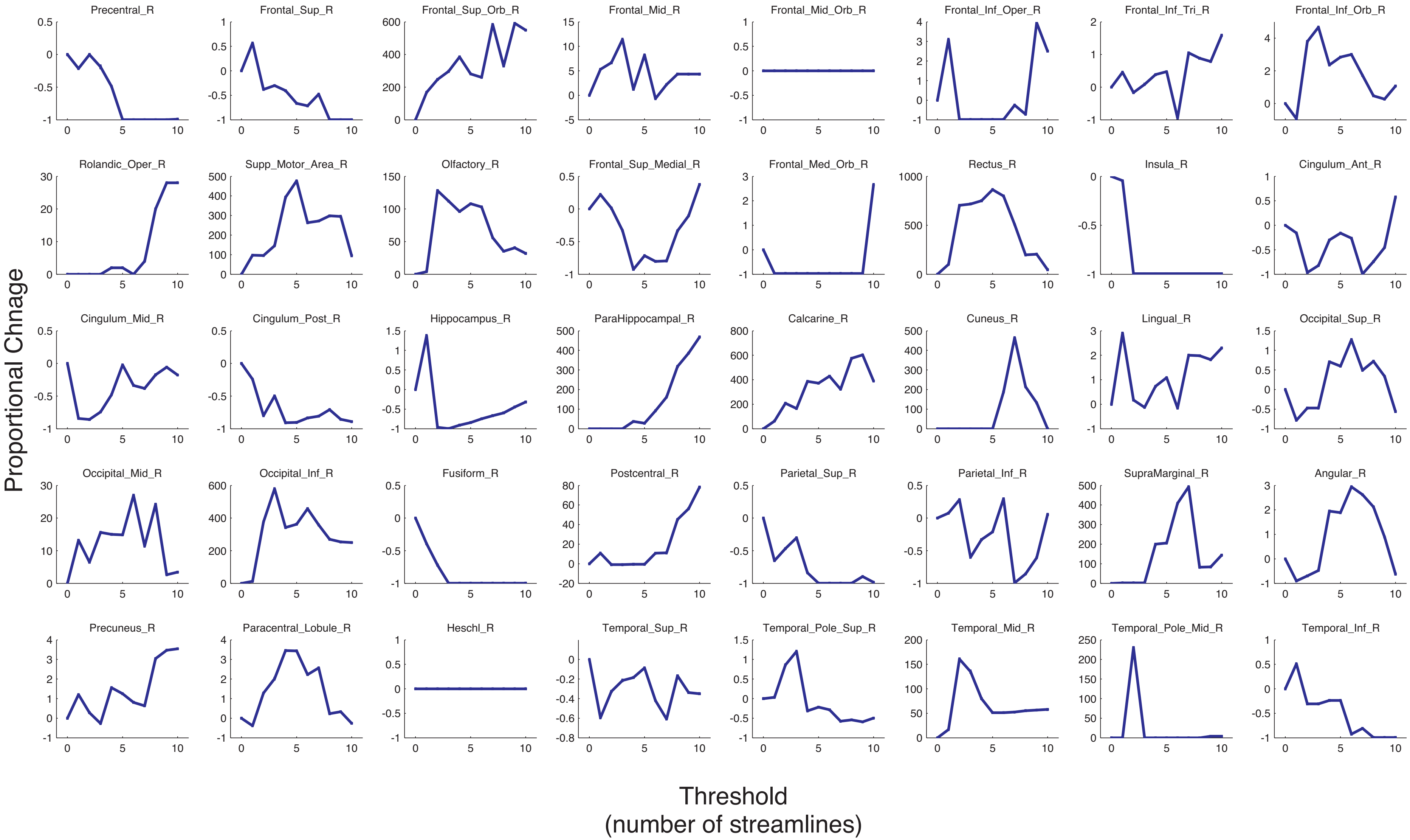

Fig. S1.10. Effects of thresholds on local betweenness.

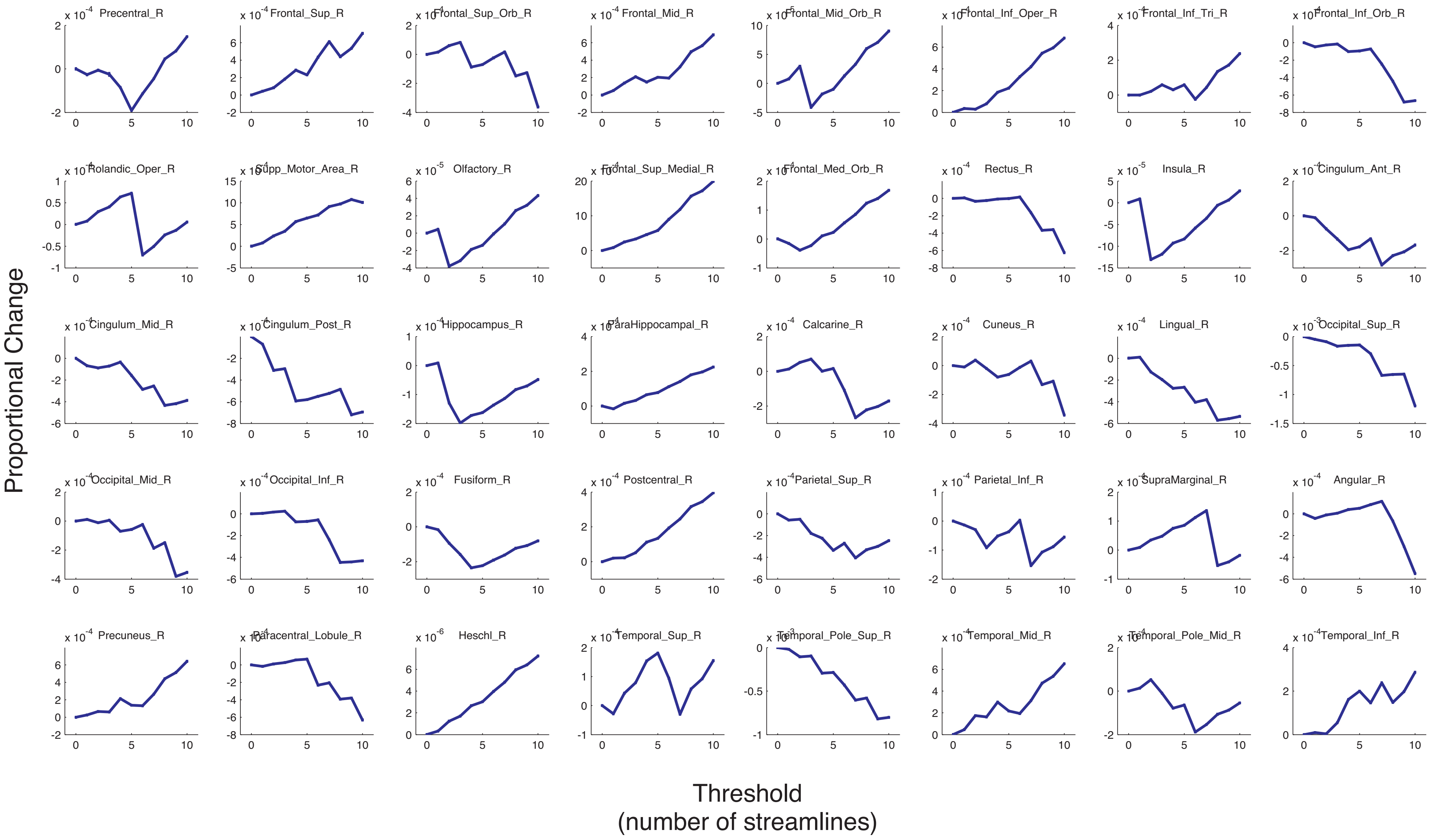

Fig. S1.11. Effects of thresholds on strength.

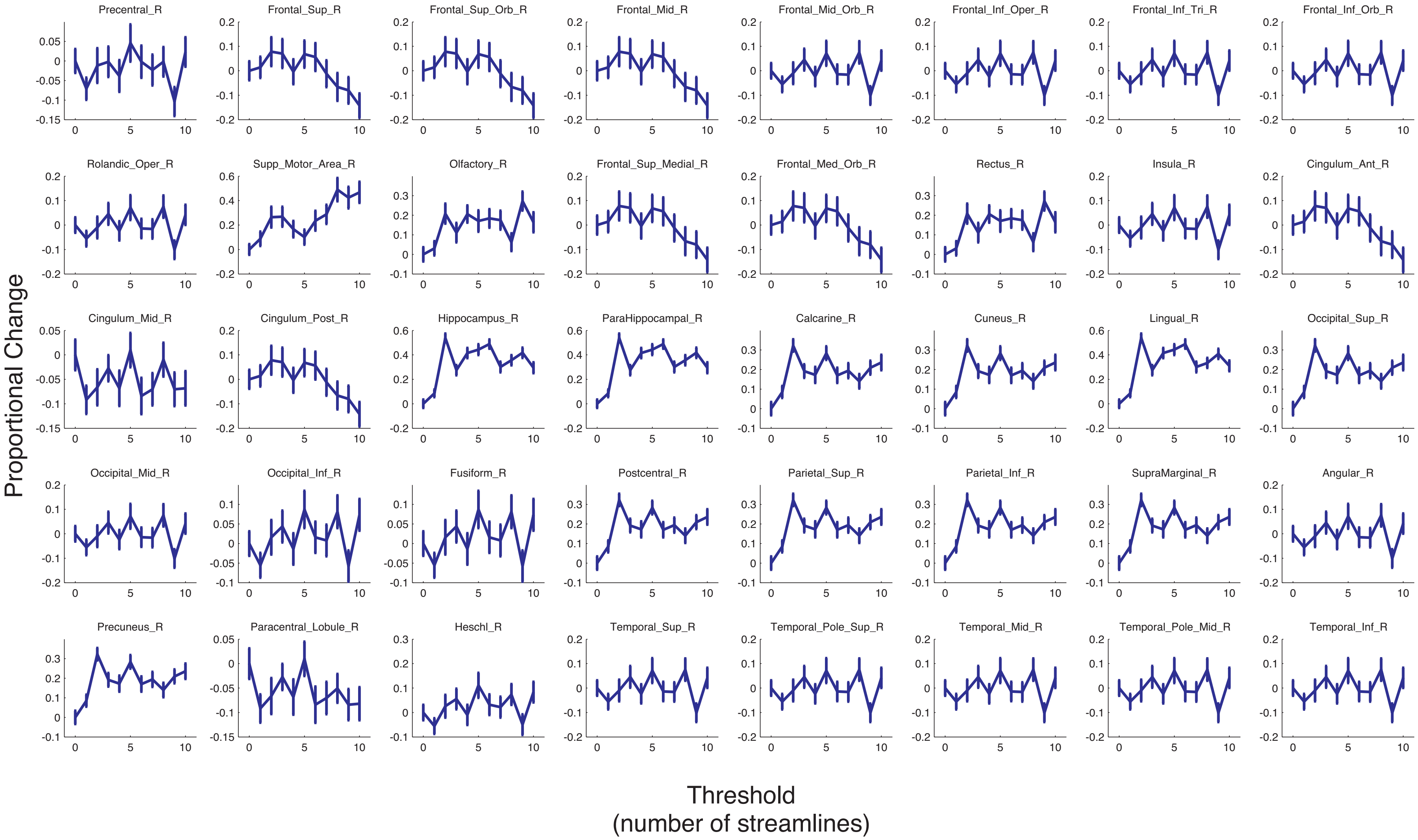

Fig. S1.12. Effects of thresholds on modularity.
