# Supplementary material for: Overcoming the effects of false positives and threshold bias in graph theoretical analyses of neuroimaging data
Source: Neuroimage. 2015 Sep;118:313–33. doi: 10.1016/j.neuroimage.2015.05.011 (PMC4558463; doi:10.1016/j.neuroimage.2015.05.011)
Supplement: Supplementary file 2 — Comparison of proportional changes in GT metric with studies of clinical groups. [file mmc2.pdf]

Supplementary material S2: Comparison of proportional changes in GT metric with studies of clinical groups.

Although the changes due to FPs observed in experiment 1a are significant. The magnitude of the effect is relatively small (in the order of about 0.03 proportional change). These values may not be deemed as evidence of a critical effect of FPs.

To examine this issue further, we summarised findings from a range of studies that have examined network topology using tractography data in various clinical disorders (table S2.1). The mean of the network metrics in the healthy and patient groups were used to estimate the mean proportional difference between the groups. Only results where a significant difference between groups (p<0.05) was reported are included.

The distribution of proportional changes is shown in figure S2.1. These show the range of values the proportional differences are in a similar range to those observed in experiment 1a. Therefore, the effects of FPs can have a critical impact on statistical inferences on network metrics, despite the seemingly small changes seen. It should also be reiterated that the FP rates used in in experiment 1a are much lower than should be expected for a typical tractography dataset.

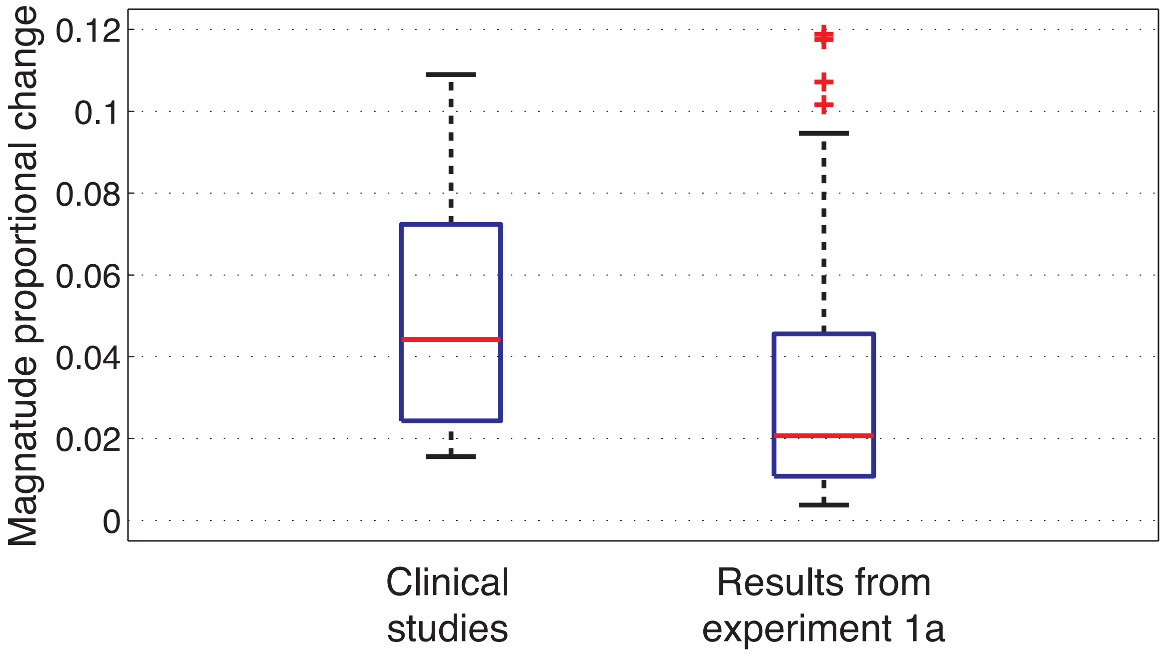

Figure S2.1. Boxplots showing the distribution of proportional differences identified from the studies summarised in table S2.1, and those observed in experiment 1a. In both cases, only changes that were significant at p<0.05 were included.

Table S2.1. Summarised results from a range of studies of network topology in clinical groups with estimate proportional change between patient and control groups. In studies where network metrics were reported for multiple thresholds, the first significant result at the lowest threshold was chosen.

Study	Disorder	Measure	Patient (mean)	Control (mean)	Proportional difference	Threshold
Zalesky et al., 2011	Schizophrenia	Deg	4.4	4.6	-0.0435	0
		GE	0.315	0.32	-0.0156	0
		nCC	5.35	4.85	0.1031	2
		nPL	1.15	1.12	0.0268	3
Lo et al., 2010	Alzheimer's disease	CPL	2.32	2.207	0.0512	
		GE	0.435	0.455	-0.0440	
Shu et al., 2011	Multiple sclerosis	GE	0.99	1.04	-0.0481	0
Leow et al., 2013	Bipolar disorder	GE	33.03	36.05	-0.0838	
		CPL	0.061	0.055	0.1091	
		CC	11.82	12.59	-0.0612	
Li et al., 2014	Autistic spectrum disorder	CC	0.176	0.162	0.0864	
		CPL	1.705	1.745	-0.0229	
(Caeyenberghs et al., 2014)	Traumatic brain Injury	GE	0.748	0.781	-0.0446	
		CPL	1.457	1.429	0.0197	
Ottet et al., 2013	22q11 deletion syndrome	GE	0.5238	0.537	-0.0249	
		CPL	2.1652	2.115	0.0237	

Deg: Degree; GE: Global efficiency; CC: clustering coefficient; CPL: Characteristic path length; nCC: Normalised clustering coefficient; nPL: Normalised path length; SW: smallworldness.

References

Caeyenberghs, K., Leemans, A., Leunissen, I., Gooijers, J., Michiels, K., Sunaert, S., Swinnen, S.P., 2014. Altered structural networks and executive deficits in traumatic brain injury patients. Brain Struct. Funct. 219, 193–209.

Leow, A., Ajilore, O., Zhan, L., Arienzo, D., Gadelkarim, J., Zhang, A., Moody, T., Van Horn, J., Feusner, J., Kumar, A., Thompson, P., Altshuler, L., 2013. Impaired inter-hemispheric integration in bipolar disorder revealed with brain network analyses. Biol. Psychiatry 73, 183–193.

Li, H., Xue, Z., Ellmore, T.M., Frye, R.E., Wong, S.T.C., 2014. Network-based analysis reveals stronger local diffusion-based connectivity and different correlations with oral language skills in brains of children with high functioning autism spectrum disorders. Hum. Brain Mapp. 35, 396–413.

Lo, C.-Y., Wang, P.-N., Chou, K.-H., Wang, J., He, Y., Lin, C.-P., 2010. Diffusion tensor tractography reveals abnormal topological organization in structural cortical networks in Alzheimer's disease. J. Neurosci. 30, 16876–16885.

Ottet, M.-C., Schaer, M., Debbané, M., Cammoun, L., Thiran, J.-P., Eliez, S., 2013. Graph theory reveals dysconnected hubs in 22q11DS and altered nodal efficiency in patients with hallucinations. Front. Hum. Neurosci. 7, 402.

Shu, N., Liu, Y., Li, K., Duan, Y., Wang, J., Yu, C., Dong, H., Ye, J., He, Y., 2011. Diffusion tensor tractography reveals disrupted topological efficiency in white matter structural networks in multiple sclerosis. Cereb. Cortex 21, 2565–77.

Zalesky, A., Fornito, A., Seal, M.L., Cocchi, L., Westin, C.-F., Bullmore, E.T., Egan, G.F., Pantelis, C., 2011. Disrupted axonal fiber connectivity in schizophrenia. Biol. Psychiatry 69, 80–9.
