# Supplementary material for: Overcoming the effects of false positives and threshold bias in graph theoretical analyses of neuroimaging data
Source: Neuroimage. 2015 Sep;118:313–33. doi: 10.1016/j.neuroimage.2015.05.011 (PMC4558463; doi:10.1016/j.neuroimage.2015.05.011)
Supplement: Supplementary file 3 — Edge-specific effects of FPs and thresholds. [file mmc3.pdf]

Supplementary material S3: Edge-specific effects of FPs and thresholds

Edge-specific effects of FPs.

Edge-specific effects of FPs in the sensitivity of global GT metrics were investigated. One FP was added systematically to each edge and the resulting GT metrics were computed. The proportional change in the GT metric of the altered network from that of the original network were computed for each edge. The variance of change for FP-NEs and FP-EEs were also computed for each node. Variance was computed across all qualifying edges on each node while non-qualifying edges were ignored.

Edge-specific proportional effects of FPs on global GT metrics for all edges are shown in figure S3.1 and variances shown in figure S3.2. Global efficiency and smallworldness showed the largest change overall with FP-NEs, while mean clustering coefficient showed relatively small changes. In particular FPs in Heschls gyrus shows a strong positive bias compared to the rest of the network, which shows a strong negative bias. Largest effects were also seen the posterior and middle cingulate regions superior frontal region, and precuneus. A similar pattern is seen for global efficiency and mean betweenness. For FP-EEs, mean betweenness showed strong effects of FP added to connections to the posterior and middle cingulate gyrus and superior occipital gyrus. There are some effects on global efficiency but other measurements show little effect.

Edge-specific effects of thresholds.

Edge-specific effects of thresholds were investigated in a similar way to edge-specific effects of FPs: Single edges were removed systematically from the ground truth network and the proportional change in GT metric from the ground truth was calculated for each node. Non-existent edges were ignored.

Edge-specific effects of thresholding are shown in figure S3.3 and variance of the effect across nodes is shown in figure S3.4. With the exception of a few regions, thresholds do not have strong edge-specific effects. Smallworldness is strongly effects by threshold applied to connections to the rolandic operculum, inferior temporal gyrus and to a lesser extent, the insula and the mid orbitofrontal cortex.

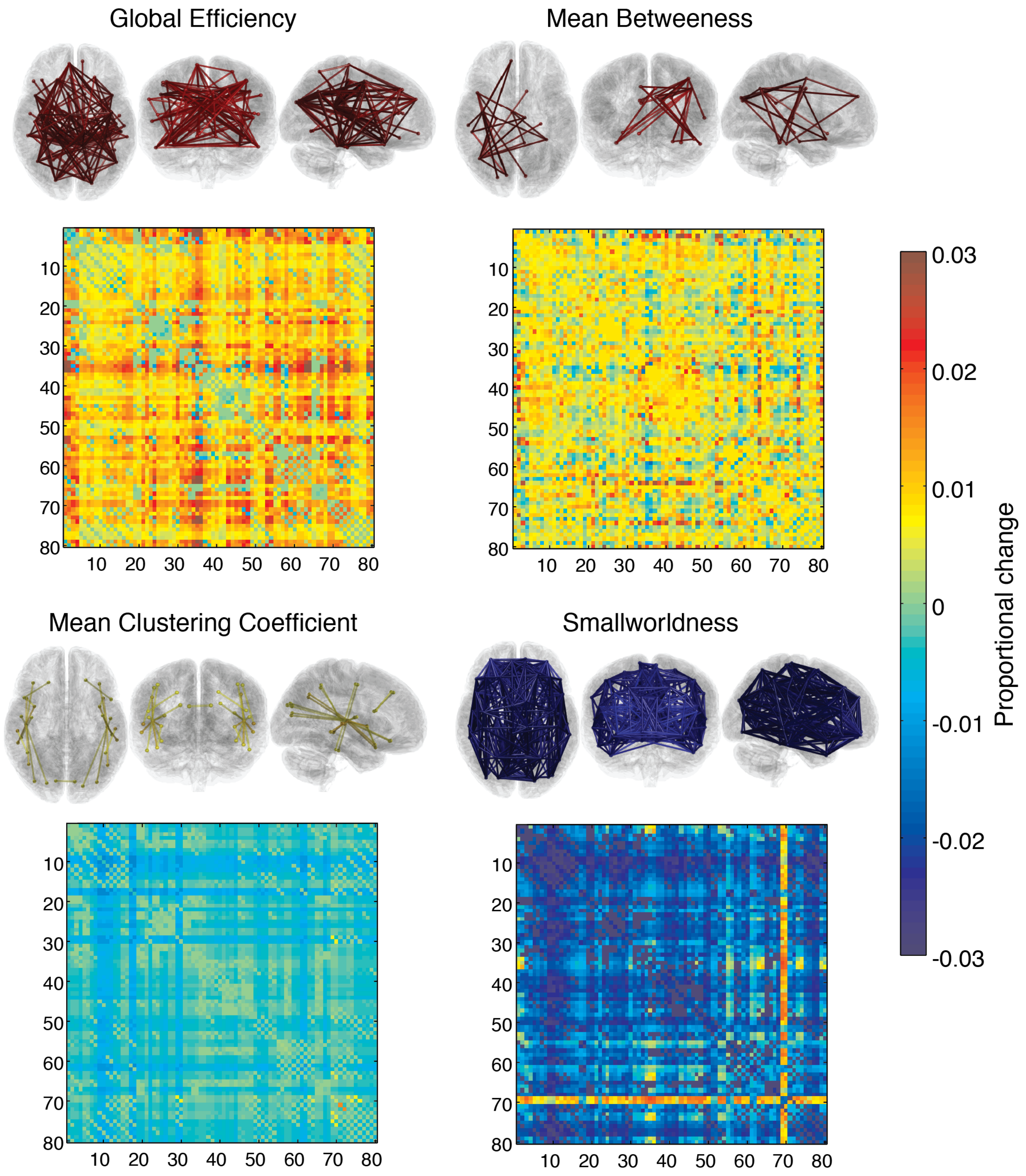

Figure S3.1. Edge-specific effects of FPs. Each matrix shows the proportional changes in GT metrics resulting from a single FP added to each edge. FPs resulting in a proportional change exceeding ±0.025 are plotted in a glass brain (except for mean clustering coefficient, where plotted edges exceed ±0.0125).

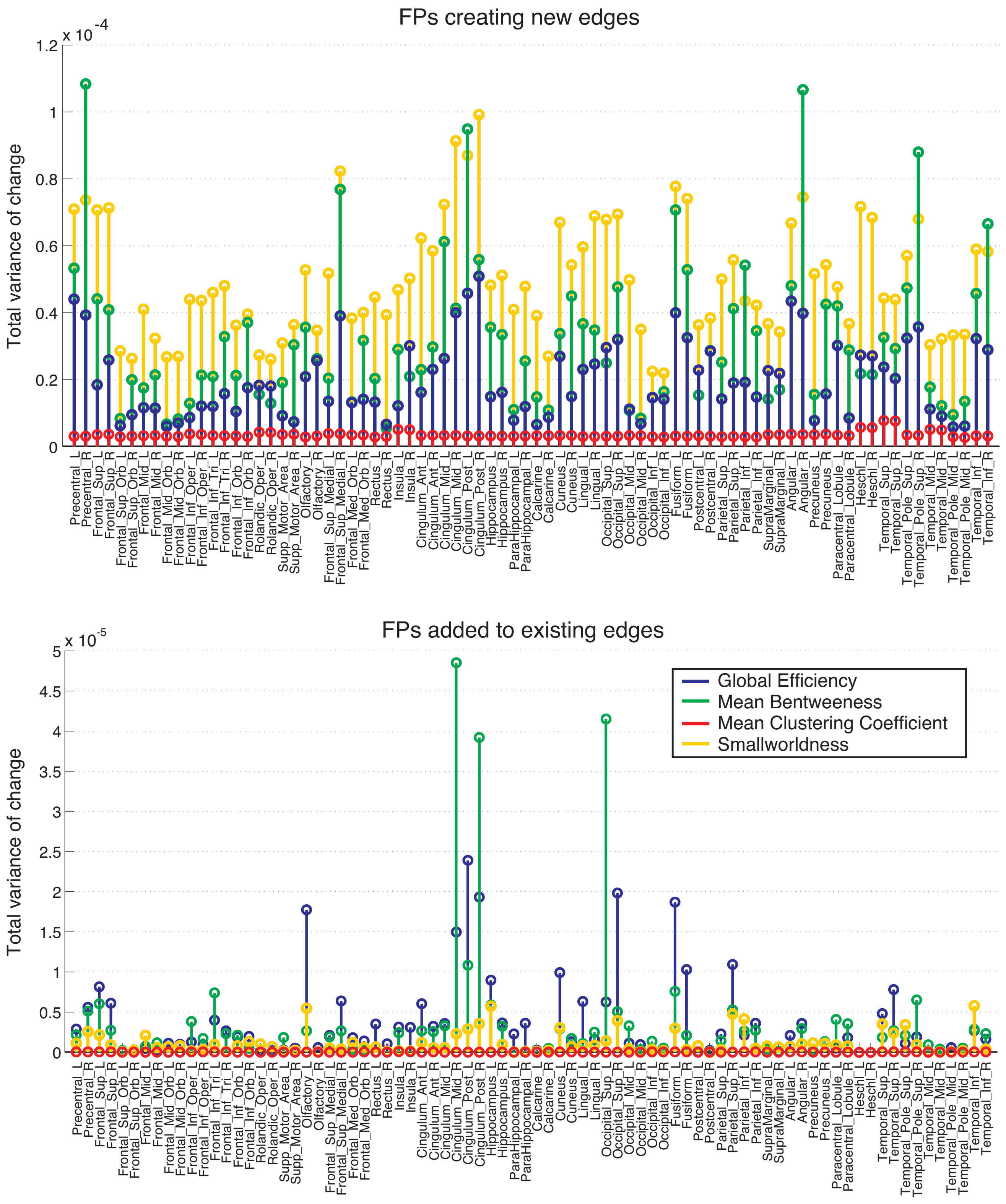

Figure S3.2. Variance of change due to FP-NEs (top) and FP-EEs (bottom) for each node. Variance was computed across all relevant edges connected to each node. Non-relevant edges were ignored.

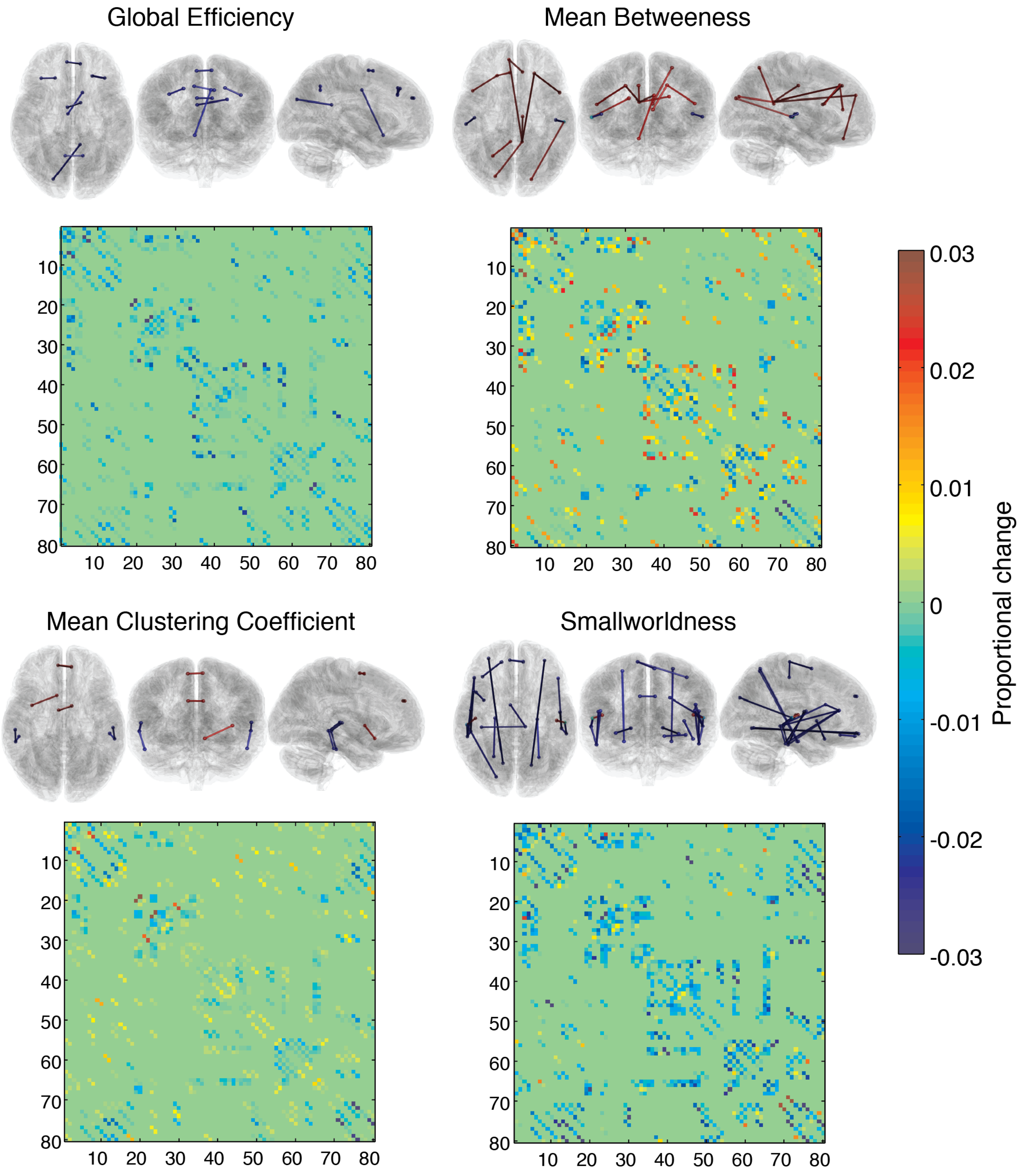

Figure S3.3. Edge-specific effects of thresholding. Each matrix shows the proportional changes in GT metrics resulting from removal of each edge. Edges whose removal result in a proportional change exceeding ±0.025 are plotted in a glass brain. Non-existent edges have zero change.

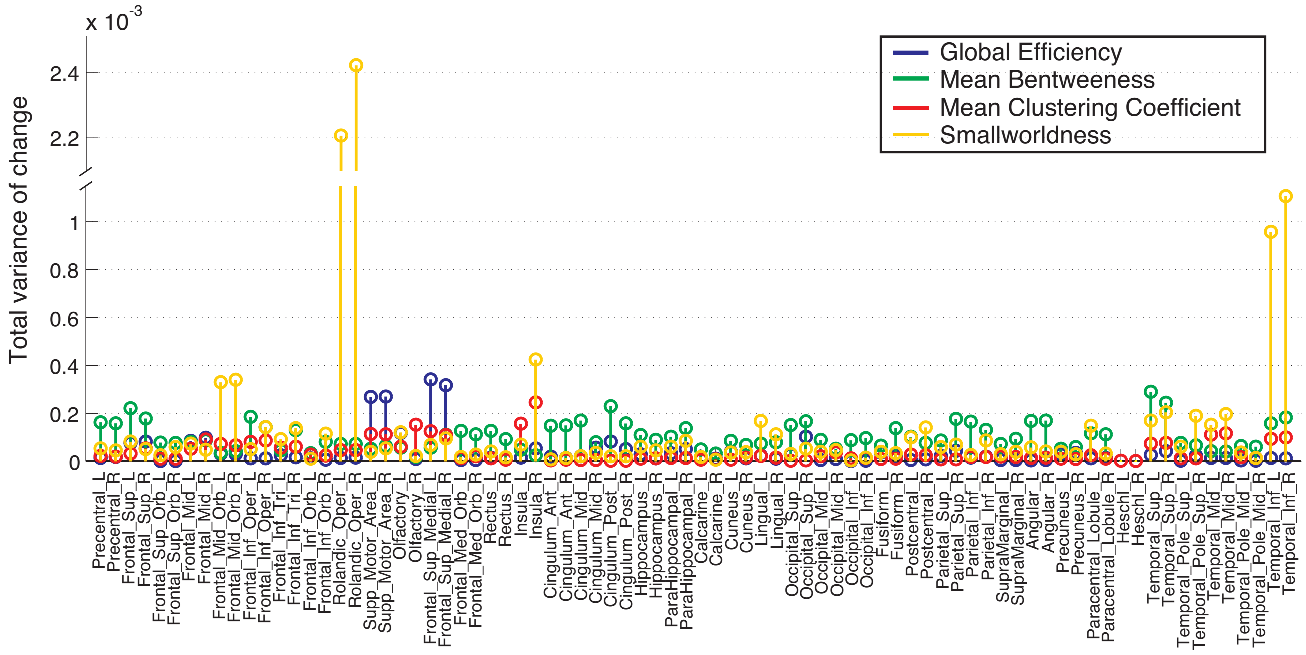

Figure S3.4. Variance of change due to thresholding for each region. Variance was computed across all relevant edges connected to each node. Non-connected edges were ignored.
