# Supplementary material for: Overcoming the effects of false positives and threshold bias in graph theoretical analyses of neuroimaging data
Source: Neuroimage. 2015 Sep;118:313–33. doi: 10.1016/j.neuroimage.2015.05.011 (PMC4558463; doi:10.1016/j.neuroimage.2015.05.011)
Supplement: Supplementary file 4 — Lillefors tests for normality. [file mmc4.pdf]

Supplementary material S4: Lillefors tests for normality

Due to the highly non-linear operations involved in GT, it is important to test the GT metrics for normality, in order to determine the appropriate statistic to employ for experiment 2. Lillefors test for normality was conducted on all GT metrics for each threshold, across the whole sample (*n*=248).

Results (figure S4.1) show all GT metrics have low, but mostly significant (at $p<0.05$) normality at low thresholds and increases at higher thresholds. The exception being mean clustering coefficient which was non-normal for thresholds of 9 to 22. Mean betweenness was also non-normal for thresholds of 4 to 6.

As the tests for normality were not consistent across metrics and thresholds, the non-parametric Mann-Whitney U-test was deemed more appropriate for statistical comparisons.

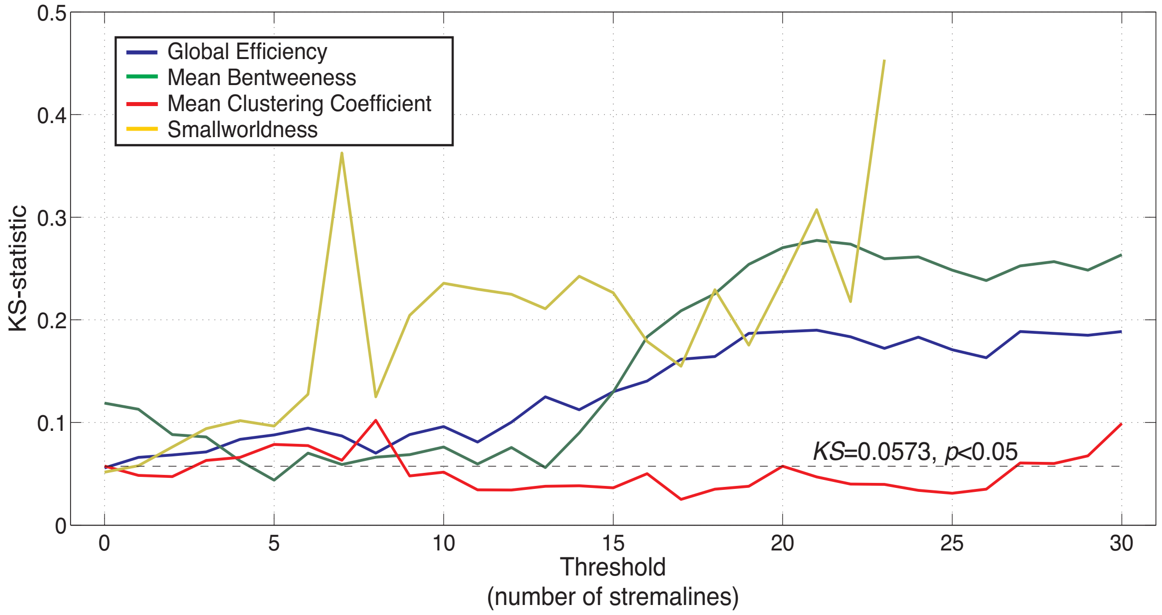

Figure S4.1. Results of Lillefors tests for each GT metric and threshold. The dotted line indicates the critical value of KS for $p<0.05$
